# Genome-Wide Meta-Analysis for Serum Calcium Identifies Significantly Associated SNPs near the Calcium-Sensing Receptor (*CASR*) Gene

**DOI:** 10.1371/journal.pgen.1001035

**Published:** 2010-07-22

**Authors:** Karen Kapur, Toby Johnson, Noam D. Beckmann, Joban Sehmi, Toshiko Tanaka, Zoltán Kutalik, Unnur Styrkarsdottir, Weihua Zhang, Diana Marek, Daniel F. Gudbjartsson, Yuri Milaneschi, Hilma Holm, Angelo DiIorio, Dawn Waterworth, Yun Li, Andrew B. Singleton, Unnur S. Bjornsdottir, Gunnar Sigurdsson, Dena G. Hernandez, Ranil DeSilva, Paul Elliott, Gudmundur I. Eyjolfsson, Jack M. Guralnik, James Scott, Unnur Thorsteinsdottir, Stefania Bandinelli, John Chambers, Kari Stefansson, Gérard Waeber, Luigi Ferrucci, Jaspal S. Kooner, Vincent Mooser, Peter Vollenweider, Jacques S. Beckmann, Murielle Bochud, Sven Bergmann

**Affiliations:** 1 Department of Medical Genetics, University of Lausanne, Lausanne, Switzerland; 2 Swiss Institute of Bioinformatics, Lausanne, Switzerland; 3 Institute of Social and Preventive Medicine, Centre Hospitalier Universitaire Vaudois and University of Lausanne, Lausanne, Switzerland; 4 National Heart and Lung Institute, Imperial College London, London, United Kingdom; 5 Clinical Research Branch, National Institute on Aging, Baltimore, Maryland, United States of America; 6 deCODE Genetics, Reykjavik, Iceland; 7 Department of Epidemiology and Public Health, Imperial College London, London, United Kingdom; 8 Department of Medicine and Sciences of Aging, Laboratory of Clinical Epidemiology, University G. d'Annunzio, Chieti, Italy; 9 Division of Genetics, GlaxoSmithKline, King of Prussia, Pennsylvania, United States of America; 10 Department of Genetics and Department of Biostatistics, University of North Carolina, Chapel Hill, North Carolina, United States of America; 11 Laboratory of Neurogenetics, National Institute on Aging, Bethesda, Maryland, United States of America; 12 The Laboratory in Mjodd, RAM, Reykjavik, Iceland; 13 Faculty of Medicine, University of Iceland, Reykjavik, Iceland; 14 Department of Endocrinology and Metabolism, University Hospital, Reykjavik, Iceland; 15 Department of Epidemiology and Biostatistics, Medical Research Council–Health Protection Agency Centre for Environment and Health, Imperial College London, London, United Kingdom; 16 Laboratory of Epidemiology, Demography, and Biometry, National Institute on Aging, Bethesda, Maryland, United States of America; 17 Geriatric Unit, Azienda Sanitaria Firenze, Florence, Italy; 18 Department of Medicine, Centre Hospitalier Universitaire Vaudois, Lausanne, Switzerland; 19 Service of Medical Genetics, Centre Hospitalier Universitaire Vaudois, Lausanne, Switzerland; University of Michigan, United States of America

## Abstract

Calcium has a pivotal role in biological functions, and serum calcium levels have been associated with numerous disorders of bone and mineral metabolism, as well as with cardiovascular mortality. Here we report results from a genome-wide association study of serum calcium, integrating data from four independent cohorts including a total of 12,865 individuals of European and Indian Asian descent. Our meta-analysis shows that serum calcium is associated with SNPs in or near the calcium-sensing receptor (*CASR*) gene on 3q13. The top hit with a p-value of 6.3×10^-37^ is rs1801725, a missense variant, explaining 1.26% of the variance in serum calcium. This SNP had the strongest association in individuals of European descent, while for individuals of Indian Asian descent the top hit was rs17251221 (p = 1.1×10^-21^), a SNP in strong linkage disequilibrium with rs1801725. The strongest locus in *CASR* was shown to replicate in an independent Icelandic cohort of 4,126 individuals (p = 1.02×10^-4)^. This genome-wide meta-analysis shows that common *CASR* variants modulate serum calcium levels in the adult general population, which confirms previous results in some candidate gene studies of the *CASR* locus. This study highlights the key role of *CASR* in calcium regulation.

## Author Summary

Calcium levels in blood serum play an important role in many biological processes. The regulation of serum calcium is under strong genetic control. This study describes the first meta-analysis of a genome-wide association study from four cohorts totaling 12,865 participants of European and Indian Asian descent. Confirming previous results in some candidate gene studies, we find that common polymorphisms at the calcium-sensing receptor (*CASR*) gene locus are associated with serum calcium concentrations. We show that *CASR* variants give rise to the strongest signals associated with serum calcium levels in both European and Indian Asian populations, while no other locus reaches genome-wide significance. Our results show that *CASR* is a key player in genetic regulation of serum calcium in the adult general population.

## Introduction

Calcium is the most abundant mineral in the human body contributing approximately one kilogram to the average adult human body mass. Whereas 99% of calcium is stored in the skeleton and teeth, the remaining 1% circulates in the bloodstream and is involved in many physiological processes including its function as a universal cellular signaling molecule [1]–[2]. Calcium plays a key role in membrane potential, which is important for muscle contraction, heart rate regulation and generation of nerve impulses. Calcium also influences bone metabolism, ion transport and many other cellular processes [3]. Approximately 2/5 of calcium in the extracellular fluid is found in blood serum. The level of serum calcium is under tight hormonal control with a normal concentration of 2.15–2.55 mmol/L. Serum calcium is under strong genetic control, with twin studies showing that the variance in total calcium due to genetic effects is between 50% and 78% [4]–[5].

While skeletal calcium is important in numerous clinical disorders, in particular bone and mineral disorders, the clinical role of serum calcium is less clear. Several [6]–[7] (but not all [8]) studies indicated that elevated serum calcium levels are associated with an increased risk of cardiovascular disease. Patients with hyperparathyroidism, who suffer from chronic hypercalcemia, have a high prevalence of hypertension and increased cardiovascular mortality [9]–[11]. However, the mechanisms underlying the putative association of serum calcium with increased cardiovascular morbidity and mortality remain unclear.

Rare monogenic forms of hypo- or hypercalcemia have been described, including disorders involving the calcium-sensing receptor (*CASR*, locus 3q13) gene. Heterozygous and homozygous *CASR* mutations that inactivate CASR are responsible, respectively, for familial hypocalciuric hypercalcemia, type 1 (also known as familial benign hypercalcemia) (OMIM #145980) [12]–[13] and neonatal severe hyperparathyroidism (OMIM #239200) [13]. On the other hand, mutations that result in CASR activation lead to autosomal dominant hypocalcemia (OMIM #146200) [14]. Mutations in many other genes have also been found to lead to disturbances of serum calcium levels (Table 1).

**Table 1:** Serum calcium candidate genes.

Gene	Gene region	Disease	OMIM	# SNPs*	Top SNP†	Top SNP p-value†
*AIRE*	chr21: 44,530,191-44,542,530	Autoimmune polyendocrine syndrome, type I	240300	870	rs2838473, rs13052277, rs717177	2.48E-04, 2.78E-05, 4.55E-03
*ALPL*	chr1: 21,581,175-21,650,208	Hypophosphatasia, infantile	241500	786	rs6426723, rs1256348, rs4654973	6.88E-03, 1.14E-03, 7.30E-04
*BSND*	chr1: 55,176,638-55,186,485	Bartter syndrome, type 4	602522	1007	rs17111592, rs11584093, rs6588528	4.61E-03, 8.40E-05, 1.70E-02
*CASR*	chr3:123,385,220-123,488,032	Familial hypocalciuric hypercalcemia, type 1; neonatal severe hyperparathyroidism; autosomal dominant hypocalcemia	145980; 239200; 146200; 241200	921	rs1801725, rs1801725, rs17251221	6.29E-37, 2.58E-18, 1.07E-21
*CDKN1B*	chr12: 12,761,576-12,766,569	Bartter syndrome, type 4	131100	855	rs3825271, rs888200, rs11055225	7.05E-04, 3.56E-03, 8.66E-04
*CLCNKA*	chr1: 16,093,672-16,105,850	Bartter syndrome, type 4	602522	545	rs12405694, rs16852052, rs6661012	1.34E-02, 2.51E-02, 1.00E-02
*CLCNKB*	chr1: 16,115,658-16,128,782	Bartter syndrome, type 3; Bartter syndrome, type 4	607364; 602522	520	rs12405694, rs16852052, rs6661012	1.34E-02, 2.51E-02, 1.00E-02
*CLDN16*	chr3: 191,588,543-191,611,035	Hypomagnesemia 3, renal	248250	1381	rs11714779, rs11714779, rs9682599	3.15E-04, 2.86E-04, 3.18E-02
*CYP27B1*	chr12: 56,442,384-56,447,145	Vitamin D-dependant rickets type I	264700	567	rs11172284, rs810204, rs715930	2.18E-03, 2.36E-03, 3.26E-02
*GATA3*	chr10: 8,136,673-8,157,170	Hypoparathyroidism; sensorineural deafness; renal disease	146255	1492	rs11812109, rs12359361, rs2765399	1.30E-03, 2.86E-03, 4.35E-03
*GCM2*	chr6: 10,981,450-10,990,084	Familial hyperparathyroidism	146200	908	rs16870899, rs16870899, rs6457160	4.88E-03, 4.59E-03, 3.36E-03
*GNAS*	chr20: 56,900,130-56,919,640	Pseudohypoparathyroidism, type IA; Pseudohypoparathyroidism, type IB	103580; 603233	949	rs2145477, rs911297, rs6015375	2.38E-04, 3.95E-03, 1.12E-02
*HRPT2*	chr1: 189,822,81-189,952,713	Hyperparathyroidism (familial isolated hyperparathyroidism); parathyroid carcinoma	145000; 608266	836	rs10737627, rs913478, rs2887613	1.15E-02, 6.50E-03, 4.71E-02
*KCNJ1*	chr11: 128,213,12-128,242,478	Bartter syndrome, antenatal, type 2	241200	1059	rs948215, rs3897566, rs7116606	1.08E-03, 1.50E-03, 3.46E-05
*MEN1*	chr11: 64,327,572-64,335,342	Hyperparathyroidism (familial isolated hyperparathyroidism); multiple endocrine neoplasia, type I	145000; 131100	498	rs7947143, rs7947143, rs11820322	1.49E-02, 6.83E-03, 2.92E-03
*PHEX*	chrX: 21,810,216-22,025,985	Hypophosphatemic rickets, X-linked dominant	307800	NA	NA	NA
*PTH*	chr11: 13,470,178-13,474,143	Familial hyperparathyroidism	146200	1292	rs10832087, rs10500780, rs1502242	9.61E-04, 1.71E-03, 1.96E-03
*PTH1R*	chr3: 46,894,240-46,920,291	Jansen's metaphyseal chondrodysplasia	156400	511	rs1402151, rs883739, rs6442037	1.03E-02, 7.54E-03, 2.18E-02
*RET*	chr10: 42,892,533-42,944,955	Multiple endocrine neoplasia, type I	131100	856	rs3026762, rs3026762, rs12265792	2.10E-04, 1.90E-04, 4.10E-02
*SLC12A1*	chr15: 46,285,790-46,383,568	Bartter syndrome, antenatal, type I	601678	925	rs1025759, rs596942, rs919129	2.25E-03, 1.29E-02, 2.30E-04
*SLC4A1*	chr17: 39,682,566-39,700,993	Renal tubular acidosis, distal, autosomal dominant	179800	494	rs12602991, rs12602991, rs708384	2.18E-03, 7.20E-03, 4.05E-03
*TBCE*	chr1: 231,856,81-231,938,321	Hypoparathyroidism-retardation-dysmorphism syndrome	241410	591	rs12133603, rs12133603, rs291353	2.34E-03, 2.18E-03, 5.38E-03
*TRPM6*	chr9: 74,566,965-74,732,564	Hypomagnesemia with secondary hypocalcemia	602014	1123	rs877809, rs877809, rs12550903	9.31E-04, 1.20E-03, 6.14E-03
*VDR*	chr12: 46,521,589-46,585,081	Vitamin D-resistant rickets type II; vitamin D-dependent rickets, type II	277440; 259700	1066	rs1859441, rs1859441, rs11168354	1.22E-03, 9.59E-04, 3.34E-04

Genes which have been shown to lead to disturbances of serum calcium levels. For each gene, we report the top SNP for the meta-analyses of all cohorts, European cohorts only and Indian Asian cohorts only.

*500 kb upstream and downstream the gene region.

†Combined, European, and Indian Asian.

In the present study, we report results obtained from meta-analysis of genome-wide associations of serum calcium levels from four cohorts with a total of 12,865 participants. We first describe the design of the study and its main finding, that variants in *CASR* give rise to the strongest signals associated with serum calcium levels in both European and Indian Asian populations. Our results confirm previous studies showing that mutations in *CASR* are associated with serum calcium levels in young healthy women [15]–[16] and extend this observation to men and women across a large spectrum of age. We show that *CASR* is a key player in the genetic regulation of serum calcium in men and women from the general adult population.

## Results

We performed a meta-analysis for genome-wide associations of serum calcium, determined by subtracting the estimated amount of calcium bound to albumin from the total serum calcium, to infer the amount of ionized calcium (see Materials and Methods). Our study included four cohorts: (i) 5404 European individuals from the Cohorte Lausanne (CoLaus) [17]–[18], (ii) 5548 European and Indian Asian individuals from the London Life Sciences Population (LOLIPOP) Study from West London UK [19]–[20], (iii) 1196 European individuals from the InCHIANTI Study (Tuscany, Italy) [21], and (iv) 717 individuals of European descent from the Baltimore Longitudinal Study of Aging (BLSA) study based in the Baltimore-Washington DC area [22], totaling 12,865 participants (see Table 2 for more detailed characteristics of each cohort).

**Table 2:** Characteristics of participants, by study.

	CoLaus	LOLIPOP European Whites	LOLIPOP Indian Asians	InCHIANTI	BLSA	deCODE
Sample size	5404	1601	3947	1196	717	4126
Gender (males/females)	2542/2862	1397/204	3832/115	533/663	390/327	1313/2813
Age(years)*	53.43(34.9,75.4)	54.5(22.6,75.0)	50.7(35.0,74.9)	68.22(21,102)	70.4(22,98)	60.1(7,103)
Pre/Post menopause	1210/1652	100/104	56/59	79/584	38/289	872/1836 (105 with pre and post measurements)
Serum calcium(mmol/L)*	2.29(0.094)	2.41(0.12)	2.37(0.11)	2.36(0.10)	2.31(0.11)	2.38(0.14)
Corrected serumCalcium (mmol/L)*	2.18(0.09)	2.31(0.09)	2.29(0.09)	2.30(0.09)	2.30(0.1)	2.31(0.14)
Serum albumin[g/L]*	44.2(2.5)	43.7(2.9)	43.4(2.9)	42.3(3.1)	40.4(3.5)	42.9(3.9)

Characteristics are shown for CoLaus, LOLIPOP European, LOLIPOP Indian Asian, InCHIANTI, BLSA and deCODE. Corrected serum calcium, designed to estimate the amount of biologically active serum calcium, is defined as Ca_corrected  =  total serum calcium [mmol/L] + (40 - albumin [g/L])/40.

*Values represent mean (range) or mean (sd).

**Figure 1:**
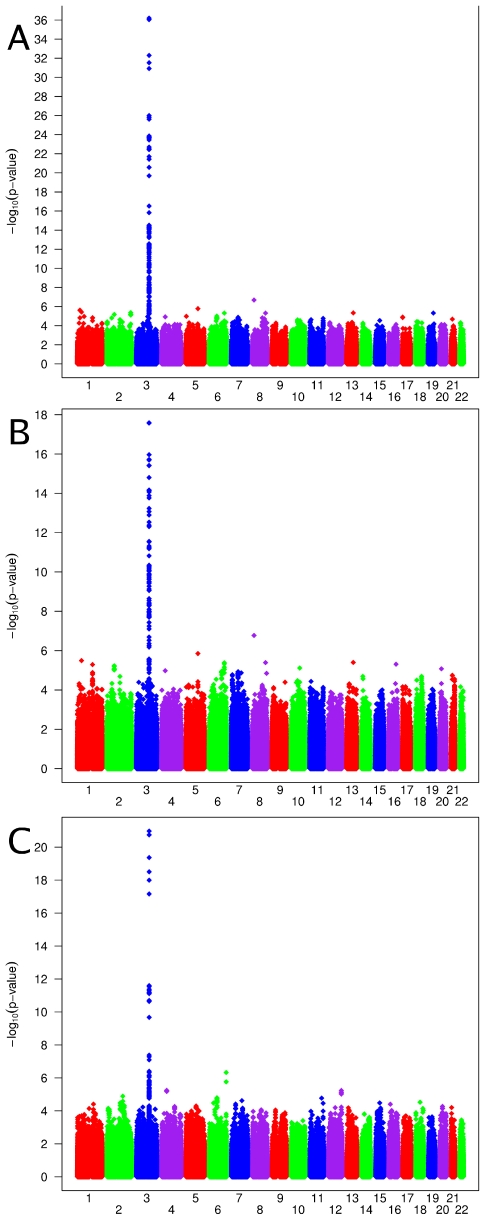
Genome-wide association results. Manhattan plots showing significance of association of all SNPs in the meta-analysis for (A) combined European and Indian Asian cohorts, (B) European cohorts only and (C) Indian Asian cohorts only. SNPs are plotted on the x-axis according to their position on each chromosome against association with serum calcium concentrations on the y-axis (shown as −log_10_ p-values).

**Figure 2:**
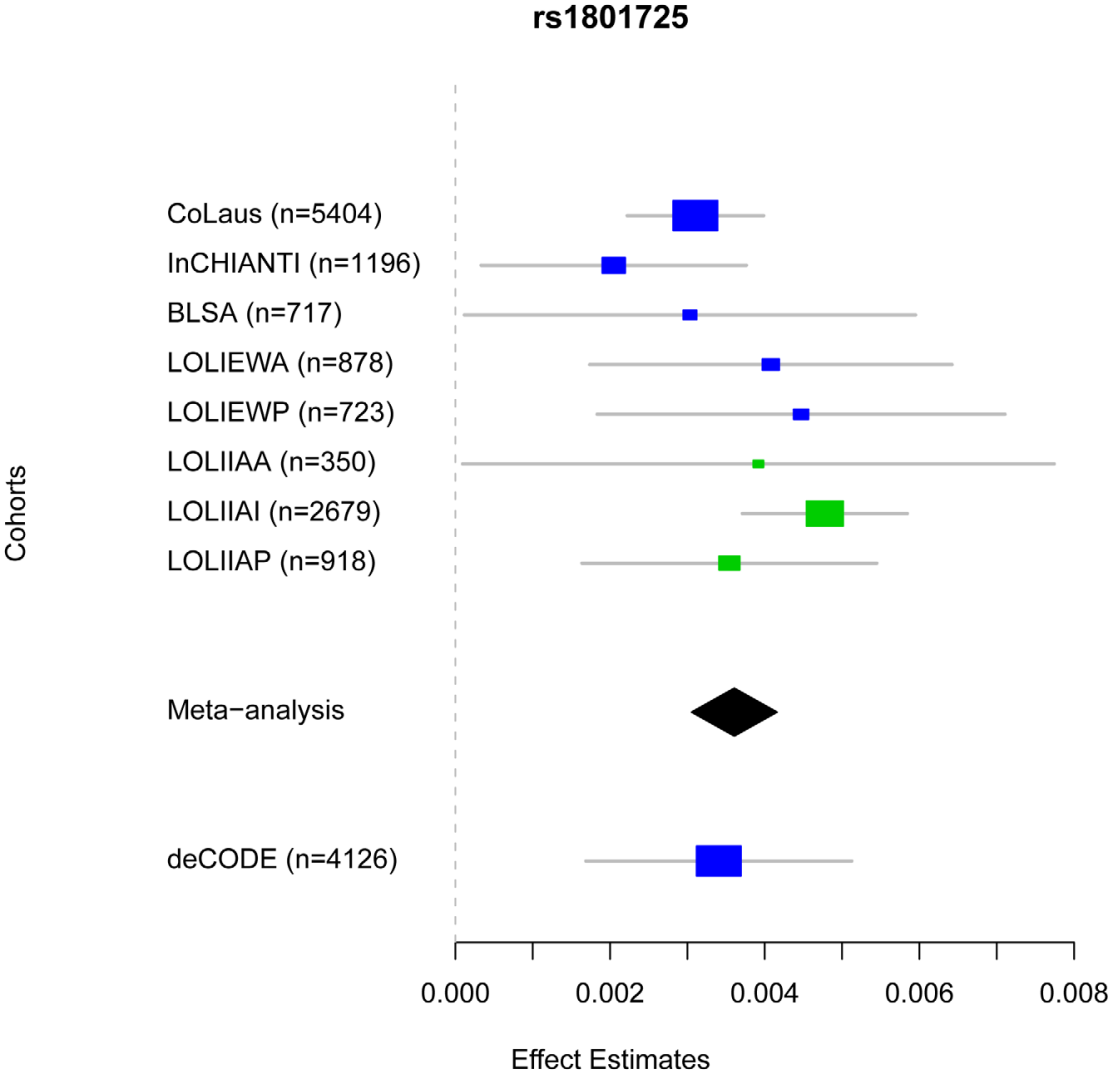
Comparison of rs1801725 significance across cohorts. The effect size and 95% confidence intervals of the serum calcium increasing *T* allele of SNP rs1801725 are shown separately for each cohort (CoLaus, LOLIPOP_EWA, LOLIPOP_EWP, LOLIPOP_IAA, LOLIPOP_IAI, LOLIPOP_IAP, BLSA, InCHIANTI) and for the replication cohort deCODE. European cohorts are shown in blue and Indian Asian cohorts are drawn in green. The size of the box is proportional to the precision 1/se^2^ and the meta-analysis estimate and 95% confidence interval across all cohorts is given by a diamond.

**Figure 3:**
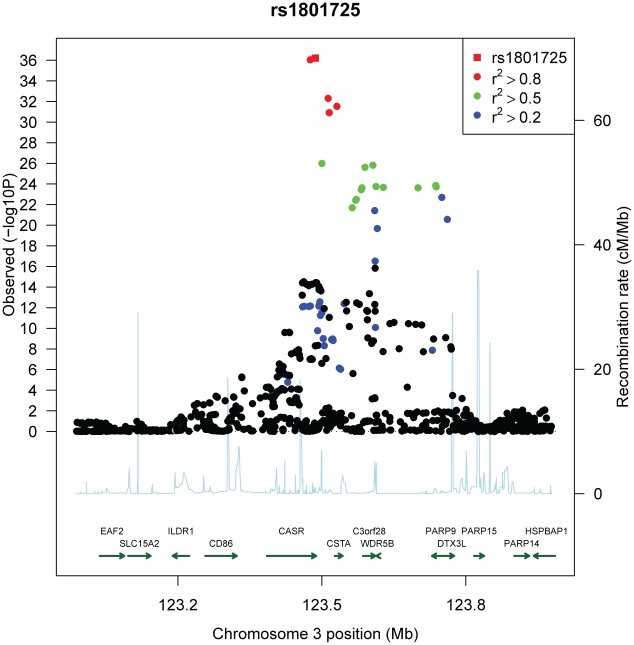
Regional association plot of the *CASR* locus. Plots show genomic position on the x-axis and −log_10_ p-values on the y-axis for SNPs in the *CASR* locus on chromosome 3. The sentinel hit is shown as a red square. Patterns of linkage disequilibrium between the sentinel SNP and all other SNPs are color-coded. Red circles indicate high correlation (r^2^>0.8), green circles indicate moderate correlation (r^2^>0.5), blue circles indicate low correlation (r^2^>0.2) and black circles indicate no correlation (r^2^<0.2). The fine-scale recombination rates from [72]–[73] are plotted in light blue.

Genome-wide association scans were performed first independently for each cohort using linear regression and then the effect sizes from each cohort were meta-analyzed (see Materials and Methods). Due to the possibility of population substructure obscuring effects of genetic variants, meta-analysis was performed separately for (i) combined European and Indian Asian cohorts (N = 12,865) and restricted to cohorts of (ii) European (N = 8,919), and (iii) Indian Asian descent (N = 3,947). The meta-analyses yielded 100 SNPs from the combined cohorts, 70 SNPs when restricting to European cohorts and 22 SNPs restricting to Indian Asian cohorts that exceeded the genome-wide significance threshold of 5×10^-8^ (Figure 1A–1C) (the full list is provided in Table S2A, S2B, S2C). All SNPs reaching statistical significance clustered around the *CASR* locus at 3q13. The most significant SNP in the (i) combined and (ii) European meta-analyses was rs1801725 (p = 6.29×10^-37^, p = 2.58×10^-18^, respectively) and in the (iii) Indian Asian meta-analysis was rs17251221 (p = 1.07×10^-21^). These two SNPs are less than 11 kb apart and are in high linkage disequilibrium with each other (r^2^ = 0.946, 0.494, 1.0, 1.0 in HapMap CEU, CHB, JPT, YRI, respectively), and therefore most likely derive from the same association signal. We find that rs1801725 explains 1.26% of the variance in serum calcium, with the effect sizes and standard errors of the serum calcium increasing *T* allele in individual cohorts shown in Figure 2 and Table S3. According to our additive model, each rs1801725 *T* allele increases log_10_ serum calcium (in units mmol/L) by 3.61×10^-3^, equivalent to a multiplicative effect of 1.008 on serum calcium (see also Table S2). At an average serum calcium level of 2.25 mmol/L, each rs1801725 *T* allele yields an increase of 0.01874 mmol/L, or 21% of one standard deviation of serum calcium levels in a normal population. The regional pattern of association of SNPs around the *CASR* locus, and their linkage disequilibrium with rs1801725, are shown in Figure 3. Of note, rs1042636, which has been associated with decreased serum calcium [23], also achieved genome-wide significance with the G minor allele associated with decreased serum calcuim (p = 4.96×10^-9^). However, conditional on the rs1801725 locus, located 12 bps upstream, the rs1042636 p-value became 3.32×10^−4^, indicating that the two loci share contributions to serum calcium levels.

To confirm the rs1801725 signal, we analyzed the association pattern with serum calcium in a separate cohort. We used a subset of 4,126 Icelandic individuals from the deCODE study [24]–[25] with serum calcium measurements. We found the rs1801725 *T* allele to be strongly associated with increased serum calcium (p = 1.02×10^-4^), replicating the key meta-analysis result.

While only the *CASR* locus reached nominal genome-wide significance for association with serum calcium, the top regions with p<10^-5^ are shown in Table 3. These SNPs cover 12 regions, the significance of which is displayed across cohorts in Figure S3. There were no SNPs in other candidate genes (which have previously been shown to be involved in disorders associated with disturbed serum calcium levels) that were associated with serum calcium at genome-wide significance. The most significant SNPs within 500 kb of the gene transcripts are shown in Table 1. Considering the set of 18,611 distinct SNPs mapping to the set of serum calcium candidate genes excluding *CASR*, we find no significant association (at significance level 0.05 and applying the Bonferroni correction for multiple testing, giving a cut-off p-value of 2.69×10^-6^, see also Figure S4). Indeed, fixing the sample size and genome-wide significance threshold our study is well-powered (≥0.80) to detect SNPs explaining at least 0.31% of the variance. Therefore the common SNPs within the candidate genes (excluding CASR) likely play at best a small role in serum calcium regulation.

**Table 3:** Significance of top SNPs.

Combined European and Indian Asian Cohorts											
db SNP	Chr	Position (Build 35)	Coded Allele	Non- Coded Allele	Frequency Coded Allele	Beta	Se	Lower 95% CI	Upper 95% CI	P-value	R^2^
rs1801725	3	123486447	T	G	17.75	3.61E-03	2.81E-04	3.06E-03	4.16E-03	**6.29E-37**	0.0126
rs17120351	8	14731368	T	C	96.24	4.03E-03	7.67E-04	2.52E-03	5.53E-03	2.06E-07	0.0021
rs7448017	5	117800594	T	G	17.83	-1.73E-03	3.57E-04	-2.43E-03	-1.03E-03	1.65E-06	0.0018
rs742393	1	25271187	G	A	78.86	2.11E-03	4.44E-04	1.24E-03	2.98E-03	2.41E-06	0.0018
rs16827695	1	40956147	G	A	94.52	3.46E-03	7.41E-04	2.01E-03	4.92E-03	3.74E-06	0.0017
rs1550532	2	234046848	G	C	74.24	-1.15E-03	2.48E-04	-1.64E-03	-6.66E-04	4.28E-06	0.0017
rs261503	13	81305121	G	A	91.35	2.45E-03	5.29E-04	1.41E-03	3.49E-03	4.60E-06	0.0017
rs10119	19	50098513	G	A	73.82	-1.63E-03	3.53E-04	-2.33E-03	-9.41E-04	4.76E-06	0.0017
rs17666460	6	149298132	G	A	11.16	1.93E-03	4.18E-04	1.11E-03	2.75E-03	4.81E-06	0.0017
rs16902486	8	129024247	G	C	4.26	-2.66E-03	5.75E-04	-3.78E-03	-1.53E-03	4.83E-06	0.0017
rs17005914	2	70721826	T	C	21.4	-1.47E-03	3.24E-04	-2.11E-03	-8.38E-04	6.76E-06	0.0016
rs10455097	6	74550153	C	A	47.75	-9.58E-04	2.15E-04	-1.38E-03	-5.37E-04	9.94E-06	0.0015

European Cohorts											
db SNP	Chr	Position (Build 35)	Coded Allele	Non-Coded Allele	Frequency Coded Allele	Beta	Se	Lower 95% CI	Upper 95% CI	P-value	R^2^
rs1801725	3	123486447	T	G	16.76	3.11E-03	3.55E-04	2.41E-03	3.80E-03	**2.58E-18**	0.0085
rs17120351	8	14731368	T	C	96.24	4.03E-03	7.67E-04	2.52E-03	5.53E-03	1.69E-07	0.0031
rs7448017	5	117800594	T	G	17.83	-1.73E-03	3.57E-04	-2.43E-03	-1.03E-03	1.40E-06	0.0026
rs16827695	1	40956147	G	A	94.45	3.46E-03	7.41E-04	2.01E-03	4.92E-03	3.20E-06	0.0024
rs261503	13	81305121	G	A	91.35	2.45E-03	5.29E-04	1.41E-03	3.49E-03	3.95E-06	0.0024
rs16902486	8	129024247	G	C	4.7	-2.92E-03	6.30E-04	-4.15E-03	-1.68E-03	4.04E-06	0.0024
rs17666460	6	149298132	G	A	11.16	1.93E-03	4.18E-04	1.11E-03	2.75E-03	4.13E-06	0.0024
rs12325114	16	72861392	T	C	76.14	-1.40E-03	3.05E-04	-2.00E-03	-8.01E-04	4.86E-06	0.0024
rs6427310	1	150628843	T	C	16.29	-1.65E-03	3.60E-04	-2.35E-03	-9.41E-04	5.09E-06	0.0023
rs17005914	2	70721826	T	C	21.4	-1.47E-03	3.24E-04	-2.11E-03	-8.38E-04	5.83E-06	0.0023
rs12416668	10	83645028	T	C	94.94	-2.77E-03	6.18E-04	-3.98E-03	-1.56E-03	7.60E-06	0.0023
rs6111021	20	16221853	C	A	41.75	1.20E-03	2.67E-04	6.71E-04	1.72E-03	8.36E-06	0.002

Indian Asian Cohorts											
db SNP	Chr	Position (Build 35)	Coded Allele	Non-Coded Allele	Frequency Coded Allele	Beta	Se	Lower 95% CI	Upper 95% CI	P-value	R^2^
rs17251221	3	123475937	G	A	19.24	4.72E-03	4.86E-04	3.76E-03	5.67E-03	**1.07E-21**	0.0233
rs13203335	6	166194438	T	C	28.04	-2.91E-03	5.70E-04	-4.03E-03	-1.79E-03	4.70E-07	0.0066
rs4695355	4	48030638	G	A	40.26	1.72E-03	3.75E-04	9.89E-04	2.46E-03	5.70E-06	0.0053
rs10846917	12	124568003	T	C	59.69	1.74E-03	3.78E-04	9.98E-04	2.48E-03	5.79E-06	0.0053

We report SNPs with p-values <1E-05 filtered by distinct regions, determined by merging SNPs within 1 Mb of each other. Results are given for different cohort subsets of combined European and Indian Asian cohorts, European cohorts, and Indian Asian cohorts. The Beta and SE values represent the effect of the coded allele on log_10_ corrected serum calcium levels; R^2^ represents the fraction of variation explained by the SNP.

We analyzed the association of the top SNP with several calcium-related outcomes (coronary heart disease, myocardial infarction, hypertension, stroke, osteoarthritis, osteoporosis and kidney stones). The number of cases and controls for each outcome and each cohort is given in Table S4. Logistic regression including age and pseudosex (see Materials and Methods) as covariates did not find any significant association between rs1801725 and the calcium-related outcomes, after correcting for multiple testing (effect sizes and standard errors for the *T* allele are listed in Table S5). Power calculations show that given the sample sizes for the clinical traits above, our study has good power (≥0.80) to detect odds ratios of 1.20, 1.13, 1.77, 1.27, 1.27, 1.24 and 1.75, respectively. As the smallest p-values from calcium-related traits were for osteoarthritis and osteoporosis (bonferroni-corrected p = 0.21, 0.44, respectively), we further investigated bone density traits. None of deCODE hip bone mineral density or spine bone mineral density (N = 6657 and 6838, respectively) nor InCHIANTI total bone density, trabecular bone density, cortical bone density, cortical bone thickness or cortical bone area (N = 1196) bonferroni-adjusted p-values for eight traits were significant.

## Discussion

This genome-wide scan of 12,865 individuals revealed *CASR* as the most significant (and only genome-wide significant) locus influencing serum calcium levels. Specifically, we found evidence for a strong association of SNPs located in the *CASR* locus with serum calcium levels in both Europeans and Indian Asians. The strongest locus in *CASR* was further shown to replicate in an independent Icelandic cohort of 4,126 individuals.

The top signal (rs1801725, *2956G>T*) explains 1.26% of the variance in serum calcium. Indeed, this is similar to results from other GWAS of human height [26]–[29], body mass index [30]–[31], serum urate [32]–[34] and serum lipid concentrations [34]–[36], for which the genome-wide significant loci uncovered thus far collectively explain only a small fraction of the phenotypic variance (usually at least one order of magnitude less than the total additive genetic variance estimated from heritability studies [37]–[38]). The rs1801725 *T* allele (A986S) was associated with higher serum calcium, consistent with previous findings (see Table S6). The rs1801725 polymorphism (with *T* allele frequencies of 16.76%, 19.98% in European and Indian Asian cohorts, respectively) affects serum calcium levels of a substantial proportion of the population.

The rs1801725 polymorphism encodes a missense variant in exon 7 of the *CASR* gene, which leads to a non-conservative amino-acid change (serine substitution for alanine-986, A986S corresponding to nucleotides *2956G>T*) in the cytoplasmic tail of CASR. *In vitro* studies showed that mutations within the C-terminal tail may influence several aspects of CASR function, such as signal transduction, intracellular trafficking and cell surface expression [39]–[41]. However, PolyPhen predicts rs1801725 to be a benign substitution. It is presently unclear whether this substitution gives rise to functional variants, as functional studies have yielded conflicting results [42]–[43]. Deep sequencing of this region may help identifying the causal variants. While it is still not possible to infer a direct causal role, it is of interest to note that the SNP gives rise to an amino acid change in the C-terminal tail of CASR, a domain which plays a key role in the receptor function and may potentially influence intracellular trafficking following CASR activation by extracellular calcium.

Several studies have reported associations of A986S and nearby *CASR* mutations with various phenotypes. The A986S *CASR* polymorphism has been associated with variations in circulating calcium levels in healthy adults in some studies [15], [23], [44]–[45], but not in others [46]–[47]. The fact that the latter studies were underpowered (sample size ranging from 148 to 1252) to detect a small effect size likely explains these inconsistent results. The rs1042636 (R990G) polymorphism has been associated with the magnitude of parathyroid hormone (PTH) secretion in patients with primary hyperparathyroidism [48], and preliminary results suggest that it could influence response to cinacalcet, a calcimimetic used to treat secondary hyperparathyroidism in patients with end-stage renal disease [49]. In a meta-analysis, 986S was associated with a 49% increased risk (P = 0.002) of primary hyperparathyroidism [47], [50]–[51]. Among patients with primary hyperparathyroidism, the AGQ haplotype (i.e. 986A, 990G, 1011Q, which is associated with lower serum calcium levels and hypercalciuria [52]) was associated with increased risk, and the SRQ haplotype with decreased risk, of kidney stones [50].

*CASR* has been previously considered as a candidate gene for osteoporosis [53] and coronary heart disease as well as increased total and cardiovascular mortality [54]. In our meta-analysis, we found no significant association of rs1801725 with these calcium-related phenotypes. A recent meta-analysis focusing on effects of candidate genes on osteoporosis also reports negative results for *CASR* [55]. Furthermore, results on the association of elevated serum calcium with increased cardiovascular risk in the general population are controversial [6]–[8]. It is therefore not clear to what extent serum calcium might predict cardiovascular risk. The SNPs identified in this meta-analysis could serve as genetic instruments in future studies, such as Mendelian randomization analysis in longitudinal cohorts, to further investigate the causal effect of serum calcium on osteoporosis and on cardiovascular disease risk (see Table S5 for rs1801725 effects and standard errors).

Our meta-analysis suffers from some limitations. First, we used corrected serum calcium and not directly measured ionized serum calcium. The correlation between corrected serum calcium and ionized serum calcium varies between 0.66 and 0.87 [56]–[58]. We can hypothesize that the association of ionized serum calcium with *CASR* variants would be stronger than the one with corrected serum calcium because ionized calcium is the form physiologically active on CASR. Second, data on serum phosphate, PTH or vitamin D are not available, so that we cannot explore further these relationships. Third, sample sizes for calcium-related clinical traits were limited, many clinical traits in CoLaus were self-reported instead of clinically diagnosed, and we incur a multiple testing penalty due to the number of clinical traits posited to be associated with serum calcium. However, the major strengths of the study are the hypothesis-free nature of GWAS studies, the large sample meta-analysis and the inclusion of multiple populations.

## Materials and Methods

### Cohorts

CoLaus is a population-based sample from Lausanne, Switzerland, consisting of 5435 individuals between 35 and 75 years old (after QC) of which a subset of 5404 had available serum calcium measurements. The study design and protocols have been described previously [17]–[18]. The CoLaus study was approved by the Institutional Ethic's Committee of the University of Lausanne. The London Life Sciences Prospective Population Study (LOLIPOP) is an ongoing population-based cohort study of ∼30,000 Indian Asian and European white men and women, aged 35–75 years living in West London, United Kingdom [59]. All study participants gave written consent including for genetic studies. The LOLIPOP study is approved by the local Research Ethics Committee. The participants included in the present study are a subset of 3947 Indian Asians and 1601 Europeans from the LOLIPOP cohort study. LOLIPOP individuals are separated by origin as well as the genotyping platform, with IAA, IAI or IAP denoting Indian Asians genotyped on Affymetrix, Illumina or Perlegen platforms, respectively, and EWA and EWI denoting Europeans genotyped on Affymetrix or Illumina platforms, respectively (see Table S1). The InCHIANTI study is a population-based epidemiological study aimed at evaluating the factors that influence mobility in the older population living in the Chianti region in Tuscany, Italy. The details of the study have been previously reported [60]. Overnight fasted blood samples were taken for genomic DNA extraction, and measurement of serum calcium. For this study, 1196 subjects with serum calcium and GWAS data were analyzed. The study protocol was approved by the Italian National Institute of Research and Care of Aging Institutional Review and Medstar Research Institute (Baltimore, MD). The Baltimore longitudinal study on Aging (BLSA) study is a population-based study aimed to evaluate contributors of healthy aging in the older population residing predominantly in the Baltimore-Washington DC area [61]. Starting in 1958, participants are examined every one to four years depending on their age. Blood samples were collected for DNA extraction. This analysis focused on a subset of the participants (N = 717) of European ancestry. The BLSA has continuing approval from the Institutional Review Board (IRB) of Medstar Research Institute. Approval was obtained from local ethic committees for all studies and all participants signed informed written consent. The deCODE study consists of individuals who visited a private outpatient laboratory, the Laboratory in Mjodd, Reykjavik, Iceland between 1997 and 2008. The main referral center for this laboratory is a multispecialty medical clinic in Reykjavik (Laeknasetrid). For the serum calcium analysis we used information on 4,126 individuals with both genome-wide SNP data and measured serum calcium and serum albumin. The samples for bone density analysis have previously been described in detail [24]–[25]. For this study 6,657 individuals with total hip bone mineral density (BMD) and 6,838 individuals with lumbar spine BMD and SNP data were available for analysis. All participants gave informed consent and the study was approved by the Data Protection Commission of Iceland (DPC) and the National Bioethics Committee of Iceland.

### Clinical data

For each CoLaus participant a venous blood sample was collected under fasting conditions. Measurements were conducted using a Modular P apparatus (Roche Diagnostics, Switzerland). Total serum calcium was measured by O-cresolphtalein (2.1% – 1.5% maximum inter and intra-batch CVs); albumin was measured by bromocresol green (2.5% – 0.4%). To further characterize the identified genetic variants, we analyzed the association with several outcomes postulated to be correlated with serum calcium. Within the CoLaus study, we have questionnaire responses to queries about personal histories of osteoporosis, osteoarthritis, myocardial infarction and stroke in addition to clinical data determining hypertension status, defined as previously described [17]. The assessment of LOLIPOP study participants was carried out by a trained research nurse, during a 45 minute appointment according to a standardized protocol and with regular QC audits. An interviewer-administered questionnaire was used to collect data on medical history, family history, current prescribed medication, and cardiovascular risk factors. Physical assessment included anthropometric measurements (height, weight, waist, hip) and blood pressure. Blood was collected after an 8 hour fast for biochemical analysis, including glucose, insulin, total and HDL cholesterol and triglycerides, and whole blood was taken for DNA extraction [59]. InCHIANTI serum albumin concentrations were determined as percentage of total protein using agarose electrophoretic technique (Hydragel Protein (E) 15/30, Sebia, Issy-les-Moulineaux, France). Serum calcium was measured using calorimetric assay (Roch Diagnostic, GmbH, Mannheim, Germany) by a Roche-Hitachi autoanalyzer (The intra-assay CV and 0.9% and the inter-assay CV was 1.5%). Measures of bone density, bone dimensions and osteoporosis diagnosis were assessed by peripheral quantitative computed tomography (pQCT) using the XCT 2000 device (Stratec Medizintechnik, Pforzheim, Germany) [62]. BLSA albumin concentrations were measured by a calorimetric assay using bromocresol green (Ortho-Clinical Diagnostics). Calcium concentrations were measured by a calorimetric assay (Vitros 5,1,FS).

### Genome-wide genotyping and imputation

CoLaus participants were genotyped using Affymetrix Human Mapping 500 K Array. For the genome-wide association stage, genotyping in LOLIPOP participants was carried out using the Illumina 317 K mapping array, Affymetrix Human Mapping 500 K array, and Perlegen, 284 K platforms (Table S1). Participants of the InCHIANTI and BLSA studies were genotyped using Illumina Infinium HumanHap 550 K SNP arrays were used for genotyping [21]. Imputation of allele dosage of SNPs was performed using either MACH [63] or IMPUTE [64] with parameters and quality control filters as described in . All European cohorts imputed SNPs typed in the HapMap CEU population; LOLIPOP Indian Asian cohort imputed SNPs using mixed HapMap populations, given that this showed greater concordance with real genotypes compared with use of any one HapMap population. SNPs were excluded if cohort-specific imputation quality as assessed by r2.hat (MACH) or .info (IMPUTE) metrics were <0.30. In total, 2,557,252 genotyped or imputed SNPs had data from one or more cohorts and were analyzed. Genotypes in deCODE were measured using either humanHap300, humanHap300-duo or humanCNV370.

### Statistical analysis

#### Individual genome-wide association analysis

Biologically active serum calcium is estimated by the correction, Ca_corrected  =  total serum calcium [mmol/L] + (40 - albumin [g/L])/40. Individuals with values <1.9 or >3 were removed as these were extreme outliers. Linear-regression analyses were carried out using an additive genetic model on log10-transformed corrected calcium levels adjusted for age and pseudosex (a factor variable with three values: males, pre-menopausal females and post-menopausal females). BLSA also included the first two and LOLIPOP included the first four ancestry principal components in the regression, respectively. Regression analyses were performed with QUICKTEST [65] (CoLaus), MACH2qtl (LOLIPOP) [63] or MERLIN [66] (InCHIANTI, BLSA).

#### Meta-analysis

The results from all cohorts were combined into a fixed-effects meta-analysis using inverse variance weighting. Tests for heterogeneity were assessed using Cochran's Q statistic and the log of the related H statistic [67] after grouping LOLIPOP subsets into European and Indian subsets. For rs1801725 and rs1042636 the p-values were (0.07657, 0.1432) and (0.3450, 0.8876), respectively, indicating limited between-study variability. The analysis was implemented in R and run on a quad-core Linux machine. SNPs were reported provided they had effect size estimates in at least 2 of the 5 European cohorts, in at least 2 of the 3 Indian Asian cohorts, or in at least 3 of the 8 total cohorts. For the overall meta-analysis, residual inflation of the test statistic was corrected using genomic control [68]. The inflation factor was 1.0207 for the all combined cohorts, 1.0068 for European cohorts and 1.0286 for Indian Asian cohorts. Where reported, individual study p-values are corrected for inflation using genomic control methods for genotyped and imputed SNPs combined (inflation factors for individual studies were 1.0139 (CoLaus), 0.9891 (LOLIPOP EWA), 0.9994 (LOLIPOP EWP), 0.9967 (LOLIPOP IAA), 1.0131 (LOLIPOP IAI), 0.9985 (LOLIPOP IAP), 0.9842 (InCHIANTI), 1.0019 (BLSA)). The regional association plot (Figure 3) was created modifying a publically available R script [69]. The map of fine-scale recombination rates was downloaded from the HapMap website http://www.hapmap.org/downloads/recombination/ using Phase II HapMap data (release 21). Quantile-quantile plots of the association results are shown in Figure S1A, S1B, S1C, study-specific quantile-quantile plots are shown in Figure S2. Associations below p = 5×10^−8^ were considered genome-wide significant, which corresponds to a Bonferroni correction for the estimated one million independent common variant tests in the human genome of European individuals [70]. The analysis of osteoporosis status in CoLaus and InCHIANTI was performed using logistic regression including age and pseudosex as covariates in QUICKTEST [65]. Linkage disequilibrium was estimated from HapMap CEU (2007-01, build 35 non-redundant) genotypes. LD r^2^ statistics were estimated for SNPs within 500 kb using Haploview [71].

#### Association of rs1801725 with calcium-related outcomes

For each related trait (coronary heart disease, hypertension, kidney stones, myocardial infarction, osteoarthritis, osteoporosis and stroke) we performed a fixed-effects meta-analysis of the logistic regression coefficients. We applied the bonferroni correction to adjust for multiple testing. We performed Wald-based power calculations using a type I error of 0.05/7 and meta-analysis coefficient estimates and standard errors to estimate the sample size for each trait giving power 0.80.

## Supporting Information

Figure S1 Quantile-Quantile plots of genome-wide association results. Observed -log_10_ p-values on the y-axis are plotted against theoretical -log_10_ p-values on the x-axis resulting from each meta-analysis. Results are color-coded by chromosome. The top results largely derive from the CASR locus on chromosome 3. Results are shown separately for (A) all cohorts, (B) European cohorts and (C) Indian Asian cohorts.
(6.48 MB TIF)

Figure S2 Study-specific quantile-quantile plots. Shown are observed -log_10_ p-values plotted against expected -log_10_ p-values resulting from each single study after applying genomic control correction. The study-specific λ-values were λ = 1.0139 (CoLaus), λ = 0.9891 (LOLIPOP_EWA), λ = 0.9994 (LOLIPOP_EWP), λ = 0.9967 (LOLIPOP_IAA), λ = 1.0131 (LOLIPOP_IAI), λ = 0.9985 (LOLIPOP_IAP), λ = 0.9842 (InCHIANTI), λ = 1.0019 (BLSA). For the combined European and Indian Asian, European only and Indian Asian only meta-analyses the inflation factors were 1.0207, 1.0068, and 1.0286, respectively.
(4.36 MB TIF)

Figure S3 Comparison of significance across cohorts. The effect size and 95% confidence intervals of SNPs which do not reach genome-wide significance in the combined European and Indian Asian meta-analysis are shown separately for each cohort (CoLaus, LOLIPOP_EWA, LOLIPOP_EWP, LOLIPOP_IAA, LOLIPOP_IAI, LOLIPOP_IAP, BLSA, InCHIANTI). European cohorts are drawn in blue and Indian Asian cohorts are drawn in green. The size of the box is proportional to the precision 1/se^2^ and the meta-analysis estimate and 95% confidence interval across all cohorts is given by a diamond.
(0.38 MB TIF)

Figure S4 Candidate gene QQ-plots. For 18611 SNPs mapping to candidate genes (excluding CASR), we compare observed -log_10_ p-values to the mean quantiles of the uniform distribution. As a comparison, we randomly choose a set of genes from which we select the same number of SNPs. From 1,000 random draws we calculate the 95th percentile of -log_10_ p-values (in blue). Results are shown separately for all cohorts, European only and Indian Asian only (A–C). CoLaus permuted phenotype results comparing observed p-values to the 95th percentile of -log_10_ p-values from 100 permutations are shown in (D).
(0.41 MB TIF)

Table S1 Genotyping, imputation, and analysis procedures by study. The genotyping platforms, quality control (QC) filters applied before imputation, imputation software, number of SNPs, and genotype-phenotype association software are shown for each study.
(0.05 MB DOC)

Table S2 Complete list of genome-wide significant SNPs. Below is the list of all SNPs that exceeded the threshold of genome-wide significance (p<5×10e-8). Position is given for NCBI Build 35. Meta-analysis is performed by inverse variance weighted fixed effect regression. The coded allele is the allele to which the beta (effect) estimate refers. Results are shown separately for (A) European and Indian Asian cohorts, (B) European cohorts only, and (C) Indian Asian cohorts only.
(0.24 MB DOC)

Table S3 Significance of top SNPs by cohort. Shown are study-specific results of the SNPs with genomic control (GC) p-values <1E-05 filtered by distinct regions, determined by merging SNPs within 1 Mb of each other. Results are shown separately for (A) European and Indian Asian cohorts, (B) European cohorts, and (C) Indian Asian cohorts.
(0.08 MB DOC)

Table S4 Number of cases and controls for calcium-related outcomes. For several related phenotypes, we test the association of rs1801725 with these binary responses. Shown here are the number of cases and controls for each phenotype in each cohort and the total across cohorts.
(0.04 MB DOC)

Table S5 Logistic regression of clinical phenotypes on rs1801725. We report the effect size and standard error of the rs1801725 T allele from logistic regressions of each clinical phenotype.
(0.04 MB DOC)

Table S6 Studies of CASR mutations and serum calcium. A survey of previous studies which investigate the relationship between CASR mutations and levels of serum calcium.
(0.05 MB DOC)

## References

[1] Carafoli E (2004). Calcium-mediated cellular signals: a story of failures.. Trends Biochem Sci.

[2] Carafoli E (2005). Calcium–a universal carrier of biological signals. Delivered on 3 July 2003 at the Special FEBS Meeting in Brussels.. FEBS J.

[3] Carafoli E (2004). The ambivalent nature of the calcium signal.. J Endocrinol Invest.

[4] Whitfield JB, Martin NG (1984). The effects of inheritance on constituents of plasma: a twin study on some biochemical variables.. Ann Clin Biochem.

[5] Williams PD, Puddey IB, Martin NG, Beilin LJ (1992). Platelet cytosolic free calcium concentration, total plasma calcium concentration and blood pressure in human twins: a genetic analysis.. Clin Sci (Lond).

[6] Leifsson BG, Ahren B (1996). Serum calcium and survival in a large health screening program.. J Clin Endocrinol Metab.

[7] Lind L, Skarfors E, Berglund L, Lithell H, Ljunghall S (1997). Serum calcium: a new, independent, prospective risk factor for myocardial infarction in middle-aged men followed for 18 years.. J Clin Epidemiol.

[8] Dhingra R, Sullivan LM, Fox CS, Wang TJ, D'Agostino RB (2007). Relations of serum phosphorus and calcium levels to the incidence of cardiovascular disease in the community.. Arch Intern Med.

[9] Palmer M, Adami HO, Bergstrom R, Jakobsson S, Akerstrom G (1987). Survival and renal function in untreated hypercalcaemia. Population-based cohort study with 14 years of follow-up.. Lancet.

[10] Lundgren E, Lind L, Palmer M, Jakobsson S, Ljunghall S (2001). Increased cardiovascular mortality and normalized serum calcium in patients with mild hypercalcemia followed up for 25 years.. Surgery.

[11] Wermers RA, Khosla S, Atkinson EJ, Grant CS, Hodgson SF (1998). Survival after the diagnosis of hyperparathyroidism: a population-based study.. Am J Med.

[12] Heath H, Odelberg S, Jackson CE, Teh BT, Hayward N (1996). Clustered inactivating mutations and benign polymorphisms of the calcium receptor gene in familial benign hypocalciuric hypercalcemia suggest receptor functional domains.. J Clin Endocrinol Metab.

[13] Pollak MR, Brown EM, Chou YH, Hebert SC, Marx SJ (1993). Mutations in the human Ca(2+)-sensing receptor gene cause familial hypocalciuric hypercalcemia and neonatal severe hyperparathyroidism.. Cell.

[14] Pollak MR, Brown EM, Estep HL, McLaine PN, Kifor O (1994). Autosomal dominant hypocalcaemia caused by a Ca(2+)-sensing receptor gene mutation.. Nat Genet.

[15] Cole DE, Peltekova VD, Rubin LA, Hawker GA, Vieth R (1999). A986S polymorphism of the calcium-sensing receptor and circulating calcium concentrations.. Lancet.

[16] Cole DE, Vieth R, Trang HM, Wong BY, Hendy GN (2001). Association between total serum calcium and the A986S polymorphism of the calcium-sensing receptor gene.. Mol Genet Metab.

[17] Firmann M, Mayor V, Marques-Vidal PM, Bochud M, Pecoud A (2008). The CoLaus study: a population-based study to investigate the epidemiology and genetic determinants of cardiovascular risk factors and metabolic syndrome.. BMC Cardiovasc Disord.

[18] Rodondi N, Cornuz J, Marques-Vidal P, Butler J, Hayoz D (2008). Aspirin use for the primary prevention of coronary heart disease: A population-based study in Switzerland.. Prev Med.

[19] Chambers JC, Elliott P, Zabaneh D, Zhang W, Li Y (2008). Common genetic variation near MC4R is associated with waist circumference and insulin resistance.. Nat Genet.

[20] Kooner JS, Chambers JC, Aguilar-Salinas CA, Hinds DA, Hyde CL (2008). Genome-wide scan identifies variation in MLXIPL associated with plasma triglycerides.. Nat Genet.

[21] Melzer D, Perry JR, Hernandez D, Corsi AM, Stevens K (2008). A genome-wide association study identifies protein quantitative trait loci (pQTLs).. PLoS Genet.

[22] Schock NW, Greulich RC, Andres R, Arenberg D, Costa PT (1984). Normal Human Aging: The Baltimore Longitudinal Study of Aging..

[23] Scillitani A, Guarnieri V, De GS, Muscarella LA, Battista C (2004). Blood ionized calcium is associated with clustered polymorphisms in the carboxyl-terminal tail of the calcium-sensing receptor.. J Clin Endocrinol Metab.

[24] Styrkarsdottir U, Halldorsson BV, Gretarsdottir S, Gudbjartsson DF, Walters GB (2009). New sequence variants associated with bone mineral density.. Nat Genet.

[25] Styrkarsdottir U, Halldorsson BV, Gretarsdottir S, Gudbjartsson DF, Walters GB (2008). Multiple genetic loci for bone mineral density and fractures.. N Engl J Med.

[26] Gudbjartsson DF, Walters GB, Thorleifsson G, Stefansson H, Halldorsson BV (2008). Many sequence variants affecting diversity of adult human height.. Nat Genet.

[27] Lettre G, Jackson AU, Gieger C, Schumacher FR, Berndt SI (2008). Identification of ten loci associated with height highlights new biological pathways in human growth.. Nat Genet.

[28] Weedon MN, Lettre G, Freathy RM, Lindgren CM, Voight BF (2007). A common variant of HMGA2 is associated with adult and childhood height in the general population.. Nat Genet.

[29] Weedon MN, Lango H, Lindgren CM, Wallace C, Evans DM (2008). Genome-wide association analysis identifies 20 loci that influence adult height.. Nat Genet.

[30] Frayling TM, Timpson NJ, Weedon MN, Zeggini E, Freathy RM (2007). A common variant in the FTO gene is associated with body mass index and predisposes to childhood and adult obesity.. Science.

[31] Loos RJ, Lindgren CM, Li S, Wheeler E, Zhao JH (2008). Common variants near MC4R are associated with fat mass, weight and risk of obesity.. Nat Genet.

[32] Doring A, Gieger C, Mehta D, Gohlke H, Prokisch H (2008). SLC2A9 influences uric acid concentrations with pronounced sex-specific effects.. Nat Genet.

[33] Li S, Sanna S, Maschio A, Busonero F, Usala G (2007). The GLUT9 gene is associated with serum uric acid levels in Sardinia and Chianti cohorts.. PLoS Genet.

[34] Wallace C, Newhouse SJ, Braund P, Zhang F, Tobin M (2008). Genome-wide association study identifies genes for biomarkers of cardiovascular disease: serum urate and dyslipidemia.. Am J Hum Genet.

[35] Sandhu MS, Waterworth DM, Debenham SL, Wheeler E, Papadakis K (2008). LDL-cholesterol concentrations: a genome-wide association study.. Lancet.

[36] Willer CJ, Sanna S, Jackson AU, Scuteri A, Bonnycastle LL (2008). Newly identified loci that influence lipid concentrations and risk of coronary artery disease.. Nat Genet.

[37] Goldstein DB (2009). Common genetic variation and human traits.. N Engl J Med.

[38] Maher B (2008). Personal genomes: The case of the missing heritability.. Nature.

[39] Bai M, Trivedi S, Brown EM (1998). Dimerization of the extracellular calcium-sensing receptor (CaR) on the cell surface of CaR-transfected HEK293 cells.. J Biol Chem.

[40] Bai M, Trivedi S, Lane CR, Yang Y, Quinn SJ (1998). Protein kinase C phosphorylation of threonine at position 888 in Ca2+o-sensing receptor (CaR) inhibits coupling to Ca2+ store release.. J Biol Chem.

[41] Gama L, Breitwieser GE (1998). A carboxyl-terminal domain controls the cooperativity for extracellular Ca2+ activation of the human calcium sensing receptor. A study with receptor-green fluorescent protein fusions.. J Biol Chem.

[42] Vezzoli G, Terranegra A, Arcidiacono T, Biasion R, Coviello D (2007). R990G polymorphism of calcium-sensing receptor does produce a gain-of-function and predispose to primary hypercalciuria.. Kidney Int.

[43] Harding B, Curley AJ, Hannan FM, Christie PT, Bowl MR (2006). Functional characterization of calcium sensing receptor polymorphisms and absence of association with indices of calcium homeostasis and bone mineral density.. Clin Endocrinol (Oxf).

[44] Kelly C, Gunn IR, Gaffney D, Devgun MS (2006). Serum calcium, urine calcium and polymorphisms of the calcium sensing receptor gene.. Ann Clin Biochem.

[45] Laaksonen MM, Outila TA, Karkkainen MU, Kemi VE, Rita HJ (2009). Associations of vitamin D receptor, calcium-sensing receptor and parathyroid hormone gene polymorphisms with calcium homeostasis and peripheral bone density in adult Finns.. J NutrigenetNutrigenomics.

[46] Bollerslev J, Wilson SG, Dick IM, Devine A, Dhaliwal SS (2004). Calcium-sensing receptor gene polymorphism A986S does not predict serum calcium level, bone mineral density, calcaneal ultrasound indices, or fracture rate in a large cohort of elderly women.. Calcif Tissue Int.

[47] Cetani F, Borsari S, Vignali E, Pardi E, Picone A (2002). Calcium-sensing receptor gene polymorphisms in primary hyperparathyroidism.. J Endocrinol Invest.

[48] Yamauchi M, Sugimoto T, Yamaguchi T, Yano S, Kanzawa M (2001). Association of polymorphic alleles of the calcium-sensing receptor gene with the clinical severity of primary hyperparathyroidism.. Clin Endocrinol (Oxf).

[49] Rothe HM, Shapiro WB, Sun WY, Chou SY (2005). Calcium-sensing receptor gene polymorphism Arg990Gly and its possible effect on response to cinacalcet HCl.. Pharmacogenet Genomics.

[50] Scillitani A, Guarnieri V, Battista C, De GS, Muscarella LA (2007). Primary hyperparathyroidism and the presence of kidney stones are associated with different haplotypes of the calcium-sensing receptor.. J Clin Endocrinol Metab.

[51] Miedlich S, Lamesch P, Mueller A, Paschke R (2001). Frequency of the calcium-sensing receptor variant A986S in patients with primary hyperparathyroidism.. Eur J Endocrinol.

[52] Vezzoli G, Tanini A, Ferrucci L, Soldati L, Bianchin C (2002). Influence of calcium-sensing receptor gene on urinary calcium excretion in stone-forming patients.. J Am Soc Nephrol.

[53] Kim KS, Kim GS, Hwang JY, Lee HJ, Park MH (2007). Single nucleotide polymorphisms in bone turnover-related genes in Koreans: ethnic differences in linkage disequilibrium and haplotype.. BMC Med Genet.

[54] Marz W, Seelhorst U, Wellnitz B, Tiran B, Obermayer-Pietsch B (2007). Alanine to serine polymorphism at position 986 of the calcium-sensing receptor associated with coronary heart disease, myocardial infarction, all-cause, and cardiovascular mortality.. J Clin Endocrinol Metab.

[55] Richards JB, Kavvoura FK, Rivadeneira F, Styrkarsdottir U, Estrada K (2009). Collaborative meta-analysis: associations of 150 candidate genes with osteoporosis and osteoporotic fracture.. Ann Intern Med.

[56] Björkman MP, Sorva AJ, Tilvis RS (1979). Calculated serum calcium is an insufficient surrogate for measured ionized calcium.. Archives of Gerontology and Geriatrics.

[57] Robertson WG, Marshall RW (1979). Calcium measurements in serum and plasma–total and ionized.. CRC Crit Rev Clin Lab Sci.

[58] Ladenson JH, Lewis JW, Boyd JC (1978). Failure of total calcium corrected for protein, albumin, and pH to correctly assess free calcium status.. J Clin Endocrinol Metab.

[59] Chambers JC, Zhang W, Li Y, Sehmi J, Wass MN (2009). Genome-wide association study identifies variants in TMPRSS6 associated with hemoglobin levels.. Nature Genetics.

[60] Ferrucci L, Bandinelli S, Benvenuti E, Di Iorio A, Macchi C (2000). Subsystems contributing to the decline in ability to walk: bridging the gap between epidemiology and geriatric practice in the InCHIANTI study.. J Am Geriatr Soc.

[61] Shock NW, Greulich RC, Arenberg D, Costa PT, Lakatta EG (1984). Normal Human Aging: The Baltimore Longitudinal Study of Aging..

[62] Russo CR, Lauretani F, Bandinelli S, Bartali B, Di Iorio A (2003). Aging bone in men and women: beyond changes in bone mineral density.. Osteoporos Int.

[63] Li Y, Willer C, Sanna S, Abecasis G (2009). Genotype imputation.. Annu Rev Genomics Hum Genet.

[64] Marchini J, Howie B, Myers S, McVean G, Donnelly P (2007). A new multipoint method for genome-wide association studies by imputation of genotypes.. Nat Genet.

[65] Johnson T, Kutalik Z (2008). QUICKTEST.

[66] Abecasis GR, Cherny SS, Cookson WO, Cardon LR (2002). Merlin–rapid analysis of dense genetic maps using sparse gene flow trees.. Nat Genet.

[67] Higgins JP, Thompson SG (2002). Quantifying heterogeneity in a meta-analysis.. Stat Med.

[68] Bacanu SA, Devlin B, Roeder K (2000). The power of genomic control.. Am J Hum Genet.

[69] Saxena R, Voight BF, Lyssenko V, Burtt NP, de Bakker PI (2007). Genome-wide association analysis identifies loci for type 2 diabetes and triglyceride levels.. Science.

[70] Dudbridge F, Gusnanto A (2008). Estimation of significance thresholds for genomewide association scans.. Genet Epidemiol.

[71] Barrett JC, Fry B, Maller J, Daly MJ (2005). Haploview: analysis and visualization of LD and haplotype maps.. Bioinformatics.

[72] McVean GA, Myers SR, Hunt S, Deloukas P, Bentley DR (2004). The fine-scale structure of recombination rate variation in the human genome.. Science.

[73] Winckler W, Myers SR, Richter DJ, Onofrio RC, McDonald GJ (2005). Comparison of fine-scale recombination rates in humans and chimpanzees.. Science.

