# Supplementary figures and images for: Genome-Wide Meta-Analysis for Serum Calcium Identifies Significantly Associated SNPs near the Calcium-Sensing Receptor (CASR) Gene
Source: PLoS Genet. 2010 Jul 22;6(7):e1001035. doi: 10.1371/journal.pgen.1001035 (PMC2908705; doi:10.1371/journal.pgen.1001035)

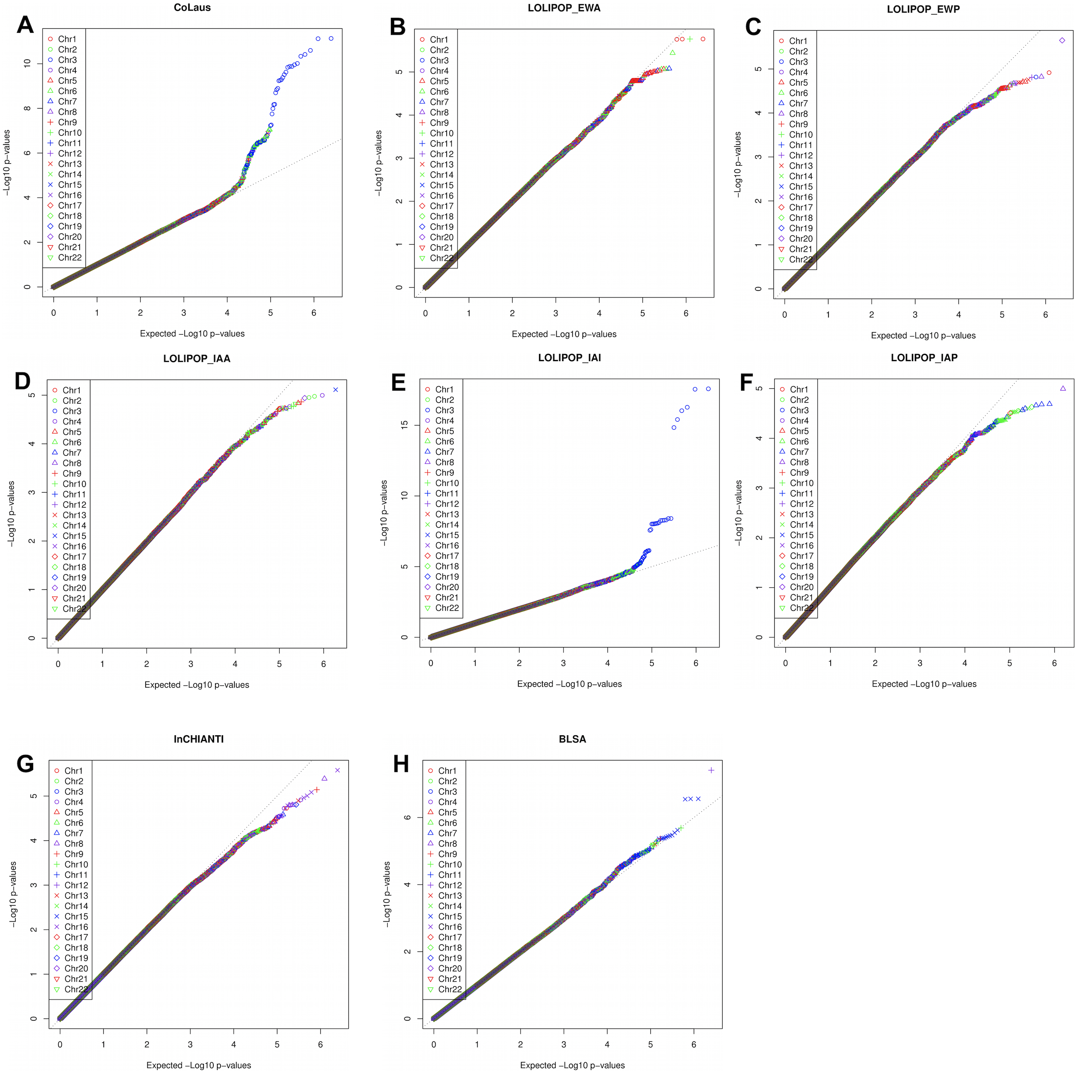

Supplement: Figure S2 — Study-specific quantile-quantile plots. Shown are observed -log10 p-values plotted against expected -log10 p-values resulting from each single study after applying genomic control correction. The study-specific λ-values were λ = 1.0139 (CoLaus), λ = 0.9891 (LOLIPOP_EWA), λ = 0.9994 (LOLIPOP_EWP), λ = 0.9967 (LOLIPOP_IAA), λ = 1.0131 (LOLIPOP_IAI), λ = 0.9985 (LOLIPOP_IAP), λ = 0.9842 (InCHIANTI), λ = 1.0019 (BLSA). For the combined European and Indian Asian, European only and Indian Asian only meta-analyses the inflation factors were 1.0207, 1.0068, and 1.0286, respectively. (4.36 MB TIF) [file pgen.1001035.s002.tif]

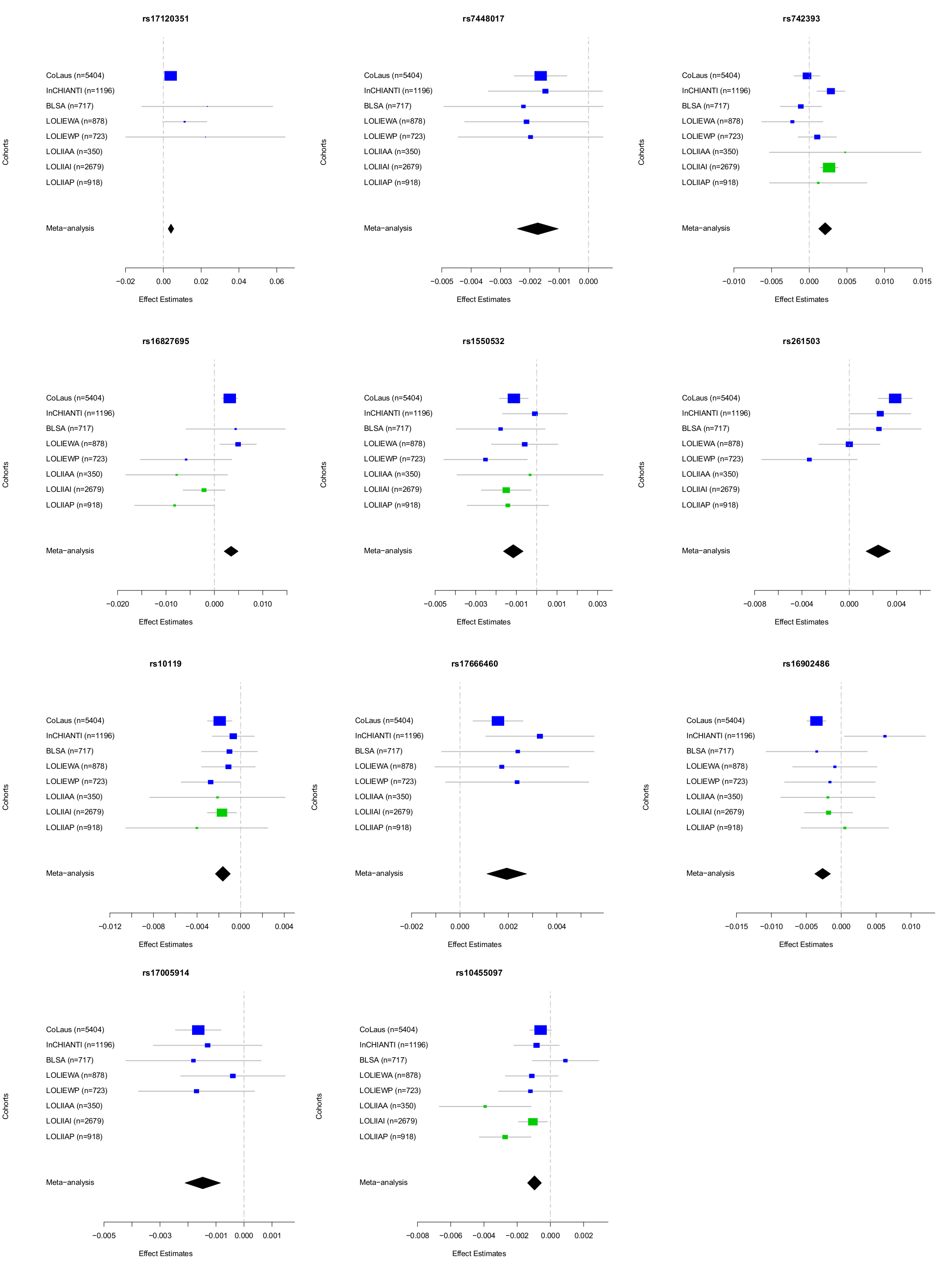

Supplement: Figure S3 — Comparison of significance across cohorts. The effect size and 95% confidence intervals of SNPs which do not reach genome-wide significance in the combined European and Indian Asian meta-analysis are shown separately for each cohort (CoLaus, LOLIPOP_EWA, LOLIPOP_EWP, LOLIPOP_IAA, LOLIPOP_IAI, LOLIPOP_IAP, BLSA, InCHIANTI). European cohorts are drawn in blue and Indian Asian cohorts are drawn in green. The size of the box is proportional to the precision 1/se2 and the meta-analysis estimate and 95% confidence interval across all cohorts is given by a diamond. (0.38 MB TIF) [file pgen.1001035.s003.tif]

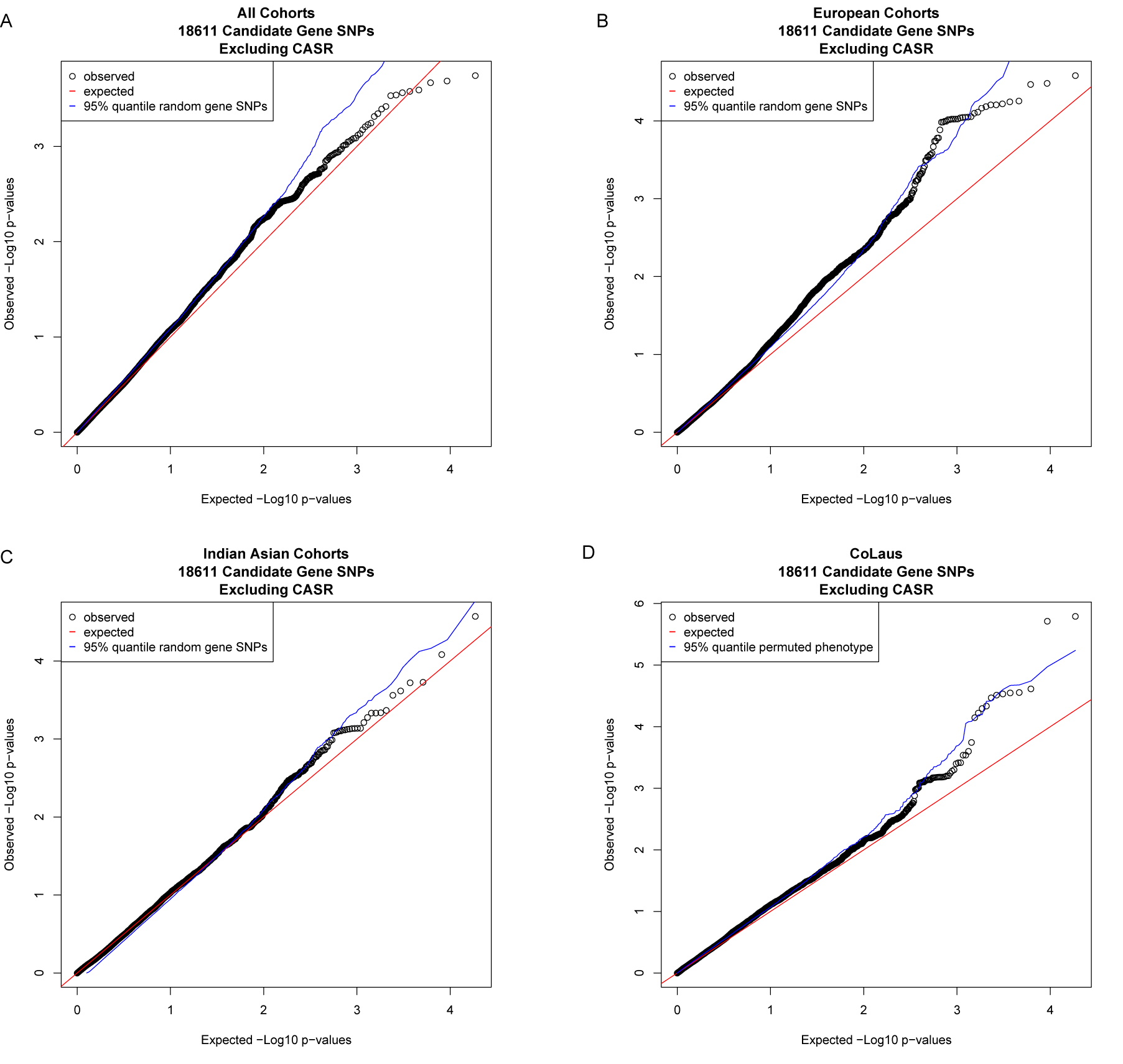

Supplement: Figure S4 — Candidate gene QQ-plots. For 18611 SNPs mapping to candidate genes (excluding CASR), we compare observed -log10 p-values to the mean quantiles of the uniform distribution. As a comparison, we randomly choose a set of genes from which we select the same number of SNPs. From 1,000 random draws we calculate the 95th percentile of -log10 p-values (in blue). Results are shown separately for all cohorts, European only and Indian Asian only (A–C). CoLaus permuted phenotype results comparing observed p-values to the 95th percentile of -log10 p-values from 100 permutations are shown in (D). (0.41 MB TIF) [file pgen.1001035.s004.tif]
