# Supplementary material for: Genome-Wide Meta-Analysis for Serum Calcium Identifies Significantly Associated SNPs near the Calcium-Sensing Receptor (CASR) Gene
Source: PLoS Genet. 2010 Jul 22;6(7):e1001035. doi: 10.1371/journal.pgen.1001035 (PMC2908705; doi:10.1371/journal.pgen.1001035)
Supplement: Table S1 — Genotyping, imputation, and analysis procedures by study. The genotyping platforms, quality control (QC) filters applied before imputation, imputation software, number of SNPs, and genotype-phenotype association software are shown for each study. (0.05 MB DOC) [file pgen.1001035.s005.doc]

| **Study acronym** | **Platform** | **Calling algorithm** | **Individual callrate** | **SNP callrate** | **HWE** | **MAF** | **SNPs for imputation** | **NCBI build** | **Method** | **SNPs for analysis** | **HapMap**  **sample** | **Statistical software** |
| --- | --- | --- | --- | --- | --- | --- | --- | --- | --- | --- | --- | --- |
| CoLaus | Affymetrix 500K | BRLMM | <90% | <70% | <1E-07 | <0.01 | 390631 | 35 | IMPUTE v0.2 | 2431919 | CEU (PhaseII,  release 21) | QUICKTEST |
| LOLIPOP_EWA | Affymetrix 500K | BRLMM | NA | <90% | <1E-06 | <0.01 | 374773 | 35 | MACH | 2557253 | CEU (PhaseII,  release 21) | MACH2qtl |
| LOLIPOP_EWP | Perlegen 284 | Perlegen | NA | <90% | <1E-06 | <0.01 | 184469 | 35 | MACH | 2557253 | CEU (PhaseII,  release 21) | MACH2qtl |
| LOLIPOP_IAA | Affymetrix 500K | BRLMM | NA | <90% | <1E-06 | <0.01 | 330744 | 35 | MACH | 1958376 | YRI, JPT, CHB, CEU  (PhaseII, release 21) | MACH2qtl |
| LOLIPOP_IAI | Illumina 317K | GenCall | NA | <90% | <1E-06 | <0.01 | 245892 | 35 | MACH | 1958376 | YRI, JPT, CHB, CEU  (PhaseII, release 21) | MACH2qtl |
| LOLIPOP_IAP | Perlegen 284 | Perlegen | NA | <90% | <1E-06 | <0.01 | 114169 | 35 | MACH | 1958376 | YRI, JPT, CHB, CEU  (PhaseII, release 21) | MACH2qtl |
| InCHIANTI | Illumina 550K | Beadstudio | <98.5% | <99% | <10-4 | <0.01 | 484115 | 35 | MACH | 2461088 | CEU (PhaseII,  release 21a, January 2007) | MERLIN (fastassoc) |
| BLSA | Illumina 550K | Beadstudio | <97% | <99% | <10-4 | <0.01 | 501764 | 35 | MACH | 2557252 | CEU (PhaseII,  release 21a, January 2007) | MERLIN (fastassoc) |
