# Supplementary material for: Genome-Wide Meta-Analysis for Serum Calcium Identifies Significantly Associated SNPs near the Calcium-Sensing Receptor (CASR) Gene
Source: PLoS Genet. 2010 Jul 22;6(7):e1001035. doi: 10.1371/journal.pgen.1001035 (PMC2908705; doi:10.1371/journal.pgen.1001035)
Supplement: Table S2 — Complete list of genome-wide significant SNPs. Below is the list of all SNPs that exceeded the threshold of genome-wide significance (p<5×10e-8). Position is given for NCBI Build 35. Meta-analysis is performed by inverse variance weighted fixed effect regression. The coded allele is the allele to which the beta (effect) estimate refers. Results are shown separately for (A) European and Indian Asian cohorts, (B) European cohorts only, and (C) Indian Asian cohorts only. (0.24 MB DOC) [file pgen.1001035.s006.doc]

(A)

| **db SNP** | **Chromosome** | **Position (Build 35)** | **Coded Allele** | **Non-Coded Allele** | **Beta** | **Se** | **Genomic Control P-value** |
| --- | --- | --- | --- | --- | --- | --- | --- |
| rs1801725 | 3 | 123486447 | T | G | 3.61E-03 | 2.81E-04 | 6.29E-37 |
| rs17251221 | 3 | 123475937 | G | A | 3.67E-03 | 2.87E-04 | 9.03E-37 |
| rs5008830 | 3 | 123513152 | G | A | -3.28E-03 | 2.71E-04 | 5.07E-33 |
| rs17265703 | 3 | 123531334 | G | A | 3.25E-03 | 2.72E-04 | 2.94E-32 |
| rs2001548 | 3 | 123515479 | G | A | -3.23E-03 | 2.73E-04 | 1.20E-31 |
| rs16832956 | 3 | 123500198 | G | C | 2.74E-03 | 2.53E-04 | 1.00E-26 |
| rs12107092 | 3 | 123606538 | T | C | 2.95E-03 | 2.74E-04 | 1.53E-26 |
| rs6791616 | 3 | 123589925 | T | C | 2.92E-03 | 2.72E-04 | 2.41E-26 |
| rs16833168 | 3 | 123737337 | T | C | 2.88E-03 | 2.78E-04 | 1.39E-24 |
| rs17200894 | 3 | 123612831 | G | C | 2.86E-03 | 2.77E-04 | 1.72E-24 |
| rs2270859 | 3 | 123738398 | G | A | -2.87E-03 | 2.78E-04 | 1.87E-24 |
| rs17201246 | 3 | 123628160 | C | A | -2.85E-03 | 2.77E-04 | 2.10E-24 |
| rs9789994 | 3 | 123700541 | T | A | -2.85E-03 | 2.77E-04 | 2.31E-24 |
| rs16833080 | 3 | 123583597 | T | C | 2.90E-03 | 2.82E-04 | 2.35E-24 |
| rs16833078 | 3 | 123582284 | G | A | 2.88E-03 | 2.81E-04 | 3.55E-24 |
| rs17267388 | 3 | 123750236 | G | A | -2.79E-03 | 2.77E-04 | 2.00E-23 |
| rs9834317 | 3 | 123572049 | T | G | 2.81E-03 | 2.80E-04 | 3.07E-23 |
| rs6438725 | 3 | 123570796 | T | C | -2.81E-03 | 2.80E-04 | 3.68E-23 |
| rs4491840 | 3 | 123563437 | G | A | -2.74E-03 | 2.78E-04 | 1.98E-22 |
| rs4678192 | 3 | 123610282 | G | A | 2.29E-03 | 2.35E-04 | 3.76E-22 |
| rs11929034 | 3 | 123761739 | G | A | -2.76E-03 | 2.88E-04 | 2.66E-21 |
| rs1067 | 3 | 123615655 | G | A | -2.51E-03 | 2.68E-04 | 2.04E-20 |
| rs4306808 | 3 | 123611156 | G | C | 3.20E-03 | 3.75E-04 | 2.98E-17 |
| rs1127343 | 3 | 123611084 | G | A | 2.02E-03 | 2.42E-04 | 1.47E-16 |
| rs6768471 | 3 | 123462394 | G | A | 1.84E-03 | 2.31E-04 | 3.04E-15 |
| rs9740 | 3 | 123487743 | G | A | -1.84E-03 | 2.32E-04 | 3.63E-15 |
| rs7646147 | 3 | 123489217 | T | C | 1.84E-03 | 2.32E-04 | 3.74E-15 |
| rs10222633 | 3 | 123459616 | G | A | 1.73E-03 | 2.19E-04 | 3.89E-15 |
| rs11922857 | 3 | 123480078 | G | A | 1.82E-03 | 2.31E-04 | 5.15E-15 |
| rs11711910 | 3 | 123468572 | T | G | 1.82E-03 | 2.31E-04 | 5.40E-15 |
| rs4678173 | 3 | 123473829 | C | A | 1.82E-03 | 2.31E-04 | 5.85E-15 |
| rs11716910 | 3 | 123470309 | G | A | -1.82E-03 | 2.31E-04 | 5.94E-15 |
| rs7644390 | 3 | 123472918 | T | A | 1.82E-03 | 2.31E-04 | 7.11E-15 |
| rs10934581 | 3 | 123493844 | T | C | -1.82E-03 | 2.32E-04 | 8.81E-15 |
| rs11720638 | 3 | 123495657 | G | C | 1.83E-03 | 2.35E-04 | 1.34E-14 |
| rs7633800 | 3 | 123494355 | G | A | 1.81E-03 | 2.33E-04 | 1.38E-14 |
| rs7633667 | 3 | 123494416 | G | C | -1.80E-03 | 2.33E-04 | 1.81E-14 |
| rs12185943 | 3 | 123498259 | G | A | -1.82E-03 | 2.37E-04 | 2.34E-14 |
| rs1986617 | 3 | 123599247 | T | A | 1.81E-03 | 2.37E-04 | 4.24E-14 |
| rs3749203 | 3 | 123459184 | T | C | -1.71E-03 | 2.26E-04 | 6.10E-14 |
| rs10934582 | 3 | 123496044 | G | A | -1.65E-03 | 2.24E-04 | 2.64E-13 |
| rs16833051 | 3 | 123550948 | G | A | 1.84E-03 | 2.49E-04 | 2.95E-13 |
| rs11920570 | 3 | 123572792 | G | A | 1.83E-03 | 2.48E-04 | 3.33E-13 |
| rs9856248 | 3 | 123546501 | T | C | 1.74E-03 | 2.38E-04 | 4.10E-13 |
| rs12633068 | 3 | 123578264 | G | C | -1.81E-03 | 2.48E-04 | 4.65E-13 |
| rs13320117 | 3 | 123610512 | T | C | -2.02E-03 | 2.77E-04 | 4.71E-13 |
| rs13085498 | 3 | 123494053 | T | C | 1.62E-03 | 2.22E-04 | 5.80E-13 |
| rs3792289 | 3 | 123476290 | G | A | 1.58E-03 | 2.18E-04 | 6.83E-13 |
| rs13085674 | 3 | 123494041 | G | A | -1.61E-03 | 2.22E-04 | 7.00E-13 |
| rs3749208 | 3 | 123462974 | T | C | 1.58E-03 | 2.18E-04 | 7.36E-13 |
| rs13095172 | 3 | 123472947 | T | C | 1.58E-03 | 2.18E-04 | 7.51E-13 |
| rs10934578 | 3 | 123459972 | T | G | 1.58E-03 | 2.18E-04 | 8.19E-13 |
| rs17252533 | 3 | 123504897 | T | G | -1.69E-03 | 2.36E-04 | 1.21E-12 |
| rs6438726 | 3 | 123593611 | T | C | -1.75E-03 | 2.46E-04 | 1.89E-12 |
| rs4315641 | 3 | 123551565 | T | A | 1.76E-03 | 2.48E-04 | 2.02E-12 |
| rs10511410 | 3 | 123611209 | G | C | 1.76E-03 | 2.48E-04 | 2.15E-12 |
| rs7635930 | 3 | 123595247 | G | C | 1.75E-03 | 2.46E-04 | 2.22E-12 |
| rs6438720 | 3 | 123499922 | C | A | -1.59E-03 | 2.26E-04 | 3.23E-12 |
| rs13083990 | 3 | 123497256 | T | C | -1.55E-03 | 2.23E-04 | 5.63E-12 |
| rs2001547 | 3 | 123515524 | G | A | 1.63E-03 | 2.37E-04 | 8.56E-12 |
| rs9818363 | 3 | 123594981 | T | C | 1.47E-03 | 2.15E-04 | 1.46E-11 |
| rs7648255 | 3 | 123650370 | T | A | 1.68E-03 | 2.49E-04 | 2.66E-11 |
| rs4677951 | 3 | 123641930 | G | C | -1.67E-03 | 2.50E-04 | 3.46E-11 |
| rs6769837 | 3 | 123680674 | G | A | -1.67E-03 | 2.49E-04 | 3.50E-11 |
| rs6805271 | 3 | 123695649 | T | C | 1.66E-03 | 2.49E-04 | 4.03E-11 |
| rs6780306 | 3 | 123708144 | T | C | 1.65E-03 | 2.49E-04 | 4.72E-11 |
| rs6798997 | 3 | 123557394 | G | A | 1.44E-03 | 2.19E-04 | 6.58E-11 |
| rs17266816 | 3 | 123611564 | G | A | -3.04E-03 | 4.64E-04 | 8.22E-11 |
| rs12635478 | 3 | 123491243 | C | A | -1.48E-03 | 2.29E-04 | 1.70E-10 |
| rs9811123 | 3 | 123431982 | G | A | 1.49E-03 | 2.33E-04 | 2.38E-10 |
| rs9866419 | 3 | 123422899 | G | A | 1.49E-03 | 2.33E-04 | 2.50E-10 |
| rs7618100 | 3 | 123432810 | G | C | 1.49E-03 | 2.33E-04 | 2.57E-10 |
| rs1500530 | 3 | 123758162 | T | G | 1.72E-03 | 2.77E-04 | 8.02E-10 |
| rs10755075 | 3 | 123596031 | T | A | -1.36E-03 | 2.19E-04 | 8.53E-10 |
| rs9851884 | 3 | 123502884 | G | A | -1.41E-03 | 2.28E-04 | 1.00E-09 |
| rs6780909 | 3 | 123732854 | T | G | 1.56E-03 | 2.53E-04 | 1.06E-09 |
| rs9864290 | 3 | 123522752 | T | C | -1.41E-03 | 2.29E-04 | 1.06E-09 |
| rs4678180 | 3 | 123520487 | T | C | 1.40E-03 | 2.29E-04 | 1.23E-09 |
| rs6803098 | 3 | 123523300 | T | C | -1.40E-03 | 2.29E-04 | 1.50E-09 |
| rs6768280 | 3 | 123607742 | G | A | 1.34E-03 | 2.20E-04 | 1.63E-09 |
| rs4678191 | 3 | 123603928 | T | G | 1.34E-03 | 2.23E-04 | 2.91E-09 |
| rs4678176 | 3 | 123491865 | G | A | -2.04E-03 | 3.44E-04 | 4.47E-09 |
| rs1402200 | 3 | 123505107 | G | C | 1.34E-03 | 2.27E-04 | 4.79E-09 |
| rs1042636 | 3 | 123486459 | G | A | -2.04E-03 | 3.45E-04 | 4.96E-09 |
| rs12486285 | 3 | 123768850 | G | A | 1.56E-03 | 2.66E-04 | 6.10E-09 |
| rs12487598 | 3 | 123661292 | T | C | 1.29E-03 | 2.22E-04 | 9.40E-09 |
| rs9839782 | 3 | 123769979 | T | C | 1.75E-03 | 3.03E-04 | 1.01E-08 |
| rs2173961 | 3 | 123451537 | T | G | 1.34E-03 | 2.33E-04 | 1.20E-08 |
| rs16833165 | 3 | 123730363 | T | C | -3.57E-03 | 6.22E-04 | 1.32E-08 |
| rs9875101 | 3 | 123450780 | T | C | 1.32E-03 | 2.32E-04 | 1.64E-08 |
| rs1979869 | 3 | 123445975 | T | C | 1.30E-03 | 2.29E-04 | 1.77E-08 |
| rs16833133 | 3 | 123627563 | G | A | -3.54E-03 | 6.21E-04 | 1.78E-08 |
| rs6438737 | 3 | 123711664 | T | C | -1.26E-03 | 2.22E-04 | 1.81E-08 |
| rs2134221 | 3 | 123444936 | G | A | 1.30E-03 | 2.29E-04 | 2.04E-08 |
| rs13326577 | 3 | 123447936 | T | A | -1.30E-03 | 2.30E-04 | 2.08E-08 |
| rs7635354 | 3 | 123449328 | C | A | 1.30E-03 | 2.31E-04 | 2.59E-08 |
| rs1354162 | 3 | 123436767 | T | G | -2.00E-03 | 3.56E-04 | 2.94E-08 |
| rs1463892 | 3 | 123453076 | G | A | -1.31E-03 | 2.36E-04 | 3.60E-08 |
| rs1965358 | 3 | 123452991 | G | A | 1.31E-03 | 2.35E-04 | 3.71E-08 |

(B)

| **db SNP** | **Chromosome** | **Position (Build 35)** | **Coded Allele** | **Non-Coded Allele** | **Beta** | **Se** | **Genomic Control P-value** |
| --- | --- | --- | --- | --- | --- | --- | --- |
| rs1801725 | 3 | 123486447 | T | G | 3.11E-03 | 3.55E-04 | 2.58E-18 |
| rs17251221 | 3 | 123475937 | G | A | 3.11E-03 | 3.56E-04 | 2.67E-18 |
| rs4678192 | 3 | 123610282 | G | A | 2.35E-03 | 2.83E-04 | 1.08E-16 |
| rs17265703 | 3 | 123531334 | G | A | 2.78E-03 | 3.37E-04 | 1.89E-16 |
| rs12107092 | 3 | 123606538 | T | C | 2.79E-03 | 3.39E-04 | 2.03E-16 |
| rs5008830 | 3 | 123513152 | G | A | -2.75E-03 | 3.36E-04 | 3.79E-16 |
| rs6791616 | 3 | 123589925 | T | C | 2.75E-03 | 3.37E-04 | 3.91E-16 |
| rs2001548 | 3 | 123515479 | G | A | -2.71E-03 | 3.38E-04 | 1.57E-15 |
| rs17200894 | 3 | 123612831 | G | C | 2.68E-03 | 3.43E-04 | 6.78E-15 |
| rs17201246 | 3 | 123628160 | C | A | -2.67E-03 | 3.42E-04 | 7.36E-15 |
| rs9789994 | 3 | 123700541 | T | A | -2.67E-03 | 3.42E-04 | 8.51E-15 |
| rs16833168 | 3 | 123737337 | T | C | 2.65E-03 | 3.43E-04 | 1.31E-14 |
| rs2270859 | 3 | 123738398 | G | A | -2.64E-03 | 3.43E-04 | 1.71E-14 |
| rs16833080 | 3 | 123583597 | T | C | 2.65E-03 | 3.52E-04 | 5.87E-14 |
| rs16833078 | 3 | 123582284 | G | A | 2.63E-03 | 3.51E-04 | 8.51E-14 |
| rs17267388 | 3 | 123750236 | G | A | -2.52E-03 | 3.39E-04 | 1.27E-13 |
| rs1127343 | 3 | 123611084 | G | A | 2.11E-03 | 2.88E-04 | 2.91E-13 |
| rs9834317 | 3 | 123572049 | T | G | 2.54E-03 | 3.50E-04 | 4.20E-13 |
| rs6438725 | 3 | 123570796 | T | C | -2.53E-03 | 3.49E-04 | 4.92E-13 |
| rs16832956 | 3 | 123500198 | G | C | 2.20E-03 | 3.13E-04 | 2.79E-12 |
| rs4491840 | 3 | 123563437 | G | A | -2.42E-03 | 3.46E-04 | 2.95E-12 |
| rs11929034 | 3 | 123761739 | G | A | -2.44E-03 | 3.52E-04 | 4.82E-12 |
| rs1067 | 3 | 123615655 | G | A | -2.26E-03 | 3.27E-04 | 5.62E-12 |
| rs10222633 | 3 | 123459616 | G | A | 1.81E-03 | 2.63E-04 | 6.53E-12 |
| rs13320117 | 3 | 123610512 | T | C | -2.16E-03 | 3.18E-04 | 1.51E-11 |
| rs6768471 | 3 | 123462394 | G | A | 1.80E-03 | 2.72E-04 | 4.52E-11 |
| rs4306808 | 3 | 123611156 | G | C | 3.05E-03 | 4.63E-04 | 4.92E-11 |
| rs3749203 | 3 | 123459184 | T | C | -1.79E-03 | 2.72E-04 | 5.20E-11 |
| rs17266816 | 3 | 123611564 | G | A | -3.04E-03 | 4.64E-04 | 6.09E-11 |
| rs9740 | 3 | 123487743 | G | A | -1.79E-03 | 2.73E-04 | 7.04E-11 |
| rs7646147 | 3 | 123489217 | T | C | 1.78E-03 | 2.73E-04 | 8.17E-11 |
| rs11711910 | 3 | 123468572 | T | G | 1.77E-03 | 2.72E-04 | 8.28E-11 |
| rs4678173 | 3 | 123473829 | C | A | 1.77E-03 | 2.72E-04 | 8.90E-11 |
| rs7644390 | 3 | 123472918 | T | A | 1.77E-03 | 2.72E-04 | 8.90E-11 |
| rs11716910 | 3 | 123470309 | G | A | -1.77E-03 | 2.72E-04 | 8.99E-11 |
| rs11922857 | 3 | 123480078 | G | A | 1.77E-03 | 2.72E-04 | 9.06E-11 |
| rs10934581 | 3 | 123493844 | T | C | -1.77E-03 | 2.74E-04 | 1.24E-10 |
| rs11720638 | 3 | 123495657 | G | C | 1.79E-03 | 2.77E-04 | 1.26E-10 |
| rs1986617 | 3 | 123599247 | T | A | 1.86E-03 | 2.88E-04 | 1.39E-10 |
| rs7633800 | 3 | 123494355 | G | A | 1.76E-03 | 2.74E-04 | 1.59E-10 |
| rs7633667 | 3 | 123494416 | G | C | -1.75E-03 | 2.74E-04 | 1.98E-10 |
| rs11920570 | 3 | 123572792 | G | A | 1.89E-03 | 2.97E-04 | 2.33E-10 |
| rs16833051 | 3 | 123550948 | G | A | 1.89E-03 | 2.98E-04 | 2.39E-10 |
| rs12633068 | 3 | 123578264 | G | C | -1.87E-03 | 2.96E-04 | 3.11E-10 |
| rs12185943 | 3 | 123498259 | G | A | -1.76E-03 | 2.79E-04 | 3.32E-10 |
| rs4315641 | 3 | 123551565 | T | A | 1.87E-03 | 2.97E-04 | 3.76E-10 |
| rs10511410 | 3 | 123611209 | G | C | 1.83E-03 | 2.93E-04 | 5.27E-10 |
| rs6438726 | 3 | 123593611 | T | C | -1.82E-03 | 2.94E-04 | 7.37E-10 |
| rs7635930 | 3 | 123595247 | G | C | 1.81E-03 | 2.94E-04 | 8.75E-10 |
| rs17252533 | 3 | 123504897 | T | G | -1.67E-03 | 2.78E-04 | 2.27E-09 |
| rs7648255 | 3 | 123650370 | T | A | 1.75E-03 | 2.94E-04 | 2.85E-09 |
| rs4677951 | 3 | 123641930 | G | C | -1.74E-03 | 2.94E-04 | 3.73E-09 |
| rs6769837 | 3 | 123680674 | G | A | -1.74E-03 | 2.94E-04 | 3.83E-09 |
| rs6805271 | 3 | 123695649 | T | C | 1.73E-03 | 2.94E-04 | 4.28E-09 |
| rs6780306 | 3 | 123708144 | T | C | 1.72E-03 | 2.93E-04 | 4.87E-09 |
| rs2001547 | 3 | 123515524 | G | A | 1.63E-03 | 2.78E-04 | 5.18E-09 |
| rs9839782 | 3 | 123769979 | T | C | 1.75E-03 | 3.03E-04 | 7.97E-09 |
| rs1500530 | 3 | 123758162 | T | G | 1.86E-03 | 3.24E-04 | 9.93E-09 |
| rs6780909 | 3 | 123732854 | T | G | 1.69E-03 | 2.95E-04 | 1.01E-08 |
| rs16833165 | 3 | 123730363 | T | C | -3.57E-03 | 6.22E-04 | 1.05E-08 |
| rs10934578 | 3 | 123459972 | T | G | 1.56E-03 | 2.73E-04 | 1.19E-08 |
| rs16833133 | 3 | 123627563 | G | A | -3.54E-03 | 6.21E-04 | 1.42E-08 |
| rs10934582 | 3 | 123496044 | G | A | -1.56E-03 | 2.75E-04 | 1.69E-08 |
| rs3749208 | 3 | 123462974 | T | C | 1.54E-03 | 2.73E-04 | 1.74E-08 |
| rs3792289 | 3 | 123476290 | G | A | 1.54E-03 | 2.73E-04 | 1.82E-08 |
| rs13095172 | 3 | 123472947 | T | C | 1.54E-03 | 2.73E-04 | 1.97E-08 |
| rs13085498 | 3 | 123494053 | T | C | 1.54E-03 | 2.74E-04 | 1.99E-08 |
| rs13085674 | 3 | 123494041 | G | A | -1.54E-03 | 2.74E-04 | 2.02E-08 |
| rs9856248 | 3 | 123546501 | T | C | 1.60E-03 | 2.90E-04 | 3.72E-08 |

(C)

| **db SNP** | **Chromosome** | **Position (Build 35)** | **Coded Allele** | **Non-Coded Allele** | **Beta** | **Se** | **Genomic**  **Control P-value** |
| --- | --- | --- | --- | --- | --- | --- | --- |
| rs17251221 | 3 | 123475937 | A | G | 4.72E-03 | 4.86E-04 | 1.07E-21 |
| rs1801725 | 3 | 123486447 | G | T | 4.45E-03 | 4.61E-04 | 1.80E-21 |
| rs5008830 | 3 | 123513152 | A | G | -4.28E-03 | 4.59E-04 | 4.31E-20 |
| rs2001548 | 3 | 123515479 | A | G | -4.19E-03 | 4.61E-04 | 3.14E-19 |
| rs17265703 | 3 | 123531334 | A | G | 4.15E-03 | 4.63E-04 | 1.02E-18 |
| rs16832956 | 3 | 123500198 | C | G | 3.76E-03 | 4.31E-04 | 6.91E-18 |
| rs16833080 | 3 | 123583597 | C | T | 3.34E-03 | 4.70E-04 | 2.52E-12 |
| rs16833078 | 3 | 123582284 | A | G | 3.34E-03 | 4.70E-04 | 2.56E-12 |
| rs4491840 | 3 | 123563437 | A | G | -3.31E-03 | 4.68E-04 | 2.87E-12 |
| rs9834317 | 3 | 123572049 | G | T | 3.29E-03 | 4.69E-04 | 4.21E-12 |
| rs6438725 | 3 | 123570796 | C | T | -3.29E-03 | 4.68E-04 | 4.24E-12 |
| rs6791616 | 3 | 123589925 | C | T | 3.22E-03 | 4.60E-04 | 4.96E-12 |
| rs12107092 | 3 | 123606538 | C | T | 3.24E-03 | 4.65E-04 | 6.24E-12 |
| rs16833168 | 3 | 123737337 | C | T | 3.31E-03 | 4.76E-04 | 6.84E-12 |
| rs2270859 | 3 | 123738398 | A | G | -3.31E-03 | 4.76E-04 | 6.87E-12 |
| rs17267388 | 3 | 123750236 | A | G | -3.33E-03 | 4.80E-04 | 7.72E-12 |
| rs17200894 | 3 | 123612831 | C | G | 3.21E-03 | 4.73E-04 | 2.00E-11 |
| rs9789994 | 3 | 123700541 | A | T | -3.20E-03 | 4.71E-04 | 2.15E-11 |
| rs11929034 | 3 | 123761739 | A | G | -3.41E-03 | 5.03E-04 | 2.18E-11 |
| rs17201246 | 3 | 123628160 | A | C | -3.20E-03 | 4.71E-04 | 2.29E-11 |
| rs1067 | 3 | 123615655 | A | G | -3.02E-03 | 4.68E-04 | 2.12E-10 |
| rs4678176 | 3 | 123491865 | A | G | -2.59E-03 | 4.65E-04 | 4.13E-08 |
