# Supplementary material for: Genome-Wide Meta-Analysis for Serum Calcium Identifies Significantly Associated SNPs near the Calcium-Sensing Receptor (CASR) Gene
Source: PLoS Genet. 2010 Jul 22;6(7):e1001035. doi: 10.1371/journal.pgen.1001035 (PMC2908705; doi:10.1371/journal.pgen.1001035)
Supplement: Table S3 — Significance of top SNPs by cohort. Shown are study-specific results of the SNPs with genomic control (GC) p-values <1E-05 filtered by distinct regions, determined by merging SNPs within 1 Mb of each other. Results are shown separately for (A) European and Indian Asian cohorts, (B) European cohorts, and (C) Indian Asian cohorts. (0.08 MB DOC) [file pgen.1001035.s007.doc]

(A)

| **db SNP** | **Chr** | **Position (Build 35)** | **GC P-value** | **CoLaus GC**  **P-value** | **LOLI_EWA GC P-value** | **LOLI_EWP GC P-value** | **LOLI_IAA GC P-value** | **LOLI_IAI GC P-value** | **LOLI_IAP GC P-value** | **BLSA GC**  **P-value** | **InCHIANTI GC P-value** |
| --- | --- | --- | --- | --- | --- | --- | --- | --- | --- | --- | --- |
| rs1801725 | 3 | 123486447 | 6.29E-37 | 7.56E-12 | 5.99E-04 | 8.86E-04 | 4.43E-02 | 2.64E-18 | 2.66E-04 | 4.17E-02 | 1.83E-02 |
| rs17120351 | 8 | 14731368 | 2.06E-07 | 7.04E-07 | 5.81E-02 | NA | NA | NA | NA | 1.89E-01 | NA |
| rs7448017 | 5 | 117800594 | 1.65E-06 | 3.93E-04 | 4.79E-02 | 1.16E-01 | NA | NA | NA | 1.09E-01 | 1.36E-01 |
| rs742393 | 1 | 25271187 | 2.41E-06 | NA | NA | 4.10E-01 | NA | 1.08E-05 | NA | 4.20E-01 | 2.16E-03 |
| rs16827695 | 1 | 40956147 | 3.74E-06 | 1.01E-04 | 1.03E-02 | NA | NA | NA | NA | 4.00E-01 | NA |
| rs1550532 | 2 | 234046848 | 4.28E-06 | 1.91E-03 | 4.76E-01 | 1.62E-02 | 8.58E-01 | 1.69E-02 | 1.63E-01 | 1.12E-01 | 9.14E-01 |
| rs261503 | 13 | 81305121 | 4.60E-06 | 1.30E-07 | 9.98E-01 | 1.00E-01 | NA | NA | NA | 1.67E-01 | 4.58E-02 |
| rs10119 | 19 | 50098513 | 4.76E-06 | 8.05E-04 | 3.65E-01 | 4.50E-02 | NA | 1.27E-02 | NA | 4.30E-01 | 4.87E-01 |
| rs17666460 | 6 | 149298132 | 4.81E-06 | 3.04E-03 | 2.18E-01 | 1.18E-01 | NA | NA | NA | 1.37E-01 | 3.54E-03 |
| rs16902486 | 8 | 129024247 | 4.83E-06 | 2.81E-07 | 7.65E-01 | 6.26E-01 | 5.80E-01 | 3.06E-01 | 8.69E-01 | 3.42E-01 | 3.30E-02 |
| rs17005914 | 2 | 70721826 | 6.76E-06 | 8.62E-05 | 6.74E-01 | 1.09E-01 | NA | NA | NA | 1.43E-01 | 1.85E-01 |
| rs10455097 | 6 | 74550153 | 9.94E-06 | 8.02E-02 | 1.63E-01 | 2.18E-01 | 5.17E-03 | 1.79E-02 | 5.65E-04 | 3.76E-01 | 2.27E-01 |

(B)

| **db SNP** | **Chr** | **Position (Build 35)** | **GC P-value** | **CoLaus GC P-value** | **LOLI_EWA GC P-value** | **LOLI_EWP GC P-value** | **BLSA GC P-value** | **InCHIANTI GC P-value** |
| --- | --- | --- | --- | --- | --- | --- | --- | --- |
| rs1801725 | 3 | 123486447 | 2.58E-18 | 7.56E-12 | 5.99E-04 | 8.86E-04 | 4.17E-02 | 1.83E-02 |
| rs17120351 | 8 | 14731368 | 1.69E-07 | 7.04E-07 | 5.81E-02 | NA | 1.89E-01 | NA |
| rs7448017 | 5 | 117800594 | 1.40E-06 | 3.93E-04 | 4.79E-02 | 1.16E-01 | 1.09E-01 | 1.36E-01 |
| rs16827695 | 1 | 40956147 | 3.20E-06 | 1.01E-04 | 1.03E-02 | NA | 4.00E-01 | NA |
| rs261503 | 13 | 81305121 | 3.95E-06 | 1.30E-07 | 9.98E-01 | 1.00E-01 | 1.67E-01 | 4.58E-02 |
| rs16902486 | 8 | 129024247 | 4.04E-06 | 2.81E-07 | 7.65E-01 | 6.26E-01 | 3.42E-01 | 3.30E-02 |
| rs17666460 | 6 | 149298132 | 4.13E-06 | 3.04E-03 | 2.18E-01 | 1.18E-01 | 1.37E-01 | 3.54E-03 |
| rs12325114 | 16 | 72861392 | 4.86E-06 | 3.44E-07 | 3.10E-01 | 9.07E-01 | 4.11E-01 | 8.46E-01 |
| rs6427310 | 1 | 150628843 | 5.09E-06 | 1.32E-04 | 2.71E-01 | 1.17E-01 | 2.81E-03 | 8.88E-01 |
| rs17005914 | 2 | 70721826 | 5.83E-06 | 8.62E-05 | 6.74E-01 | 1.09E-01 | 1.43E-01 | 1.85E-01 |
| rs12416668 | 10 | 83645028 | 7.60E-06 | 3.24E-05 | 9.14E-01 | 2.00E-01 | 2.66E-01 | 1.01E-01 |
| rs6111021 | 20 | 16221853 | 8.36E-06 | 1.60E-03 | 9.14E-01 | 2.21E-04 | 4.00E-01 | 3.20E-02 |

(C)

| **db SNP** | **Chr** | **Position (Build 35)** | **GC P-value** | **LOLI_IAA**  **GC P-value** | **LOLI_IAI**  **GC P-value** | **LOLI_IAP**  **GC P-value** |
| --- | --- | --- | --- | --- | --- | --- |
| rs17251221 | 3 | 123475937 | 1.07E-21 | 4.86E-02 | 2.82E-18 | 1.64E-04 |
| rs13203335 | 6 | 166194438 | 4.70E-07 | 9.62E-03 | 6.03E-06 | NA |
| rs4695355 | 4 | 48030638 | 5.70E-06 | 3.85E-01 | 3.44E-04 | 4.68E-03 |
| rs10846917 | 12 | 124568003 | 5.79E-06 | 1.11E-01 | 3.11E-03 | 5.98E-04 |
