# Supplementary material for: Genome-Wide Meta-Analysis for Serum Calcium Identifies Significantly Associated SNPs near the Calcium-Sensing Receptor (CASR) Gene
Source: PLoS Genet. 2010 Jul 22;6(7):e1001035. doi: 10.1371/journal.pgen.1001035 (PMC2908705; doi:10.1371/journal.pgen.1001035)
Supplement: Table S4 — Number of cases and controls for calcium-related outcomes. For several related phenotypes, we test the association of rs1801725 with these binary responses. Shown here are the number of cases and controls for each phenotype in each cohort and the total across cohorts. (0.04 MB DOC) [file pgen.1001035.s008.doc]

| **Phenotype** | **CoLaus** | | **LOLIPOP European White** | | **LOLIPOP Indian Asian** | | **InCHIANTI** | | **Total** | |
| --- | --- | --- | --- | --- | --- | --- | --- | --- | --- | --- |
|  | **Cases** | **Controls** | **Cases** | **Controls** | **Cases** | **Controls** | **Cases** | **Controls** | **Cases** | **Controls** |
| Coronary heart disease | NA | NA | 573 | 1028 | 1226 | 2721 | NA | NA | 1799 | 3749 |
| Hypertension | 1960 | 3444 | 456 | 1145 | 716 | 3231 | 877 | 319 | 4009 | 8139 |
| Kidney stones | NA | NA | NA | NA | NA | NA | 130 | 1040 | 130 | 1040 |
| Myocardial infarction | 150 | 5254 | 347 | 1254 | 252 | 3695 | 50 | 1146 | 799 | 11349 |
| Osteoarthritis | 1253 | 4151 | NA | NA | NA | NA | NA | NA | 1253 | 4151 |
| Osteoporosis | 228 | 5176 | NA | NA | NA | NA | 603 | 593 | 831 | 5769 |
| Stroke | 82 | 5322 | NA | NA | NA | NA | 56 | 1140 | 138 | 6462 |
