# Supplementary material for: Genome-Wide Meta-Analysis for Serum Calcium Identifies Significantly Associated SNPs near the Calcium-Sensing Receptor (CASR) Gene
Source: PLoS Genet. 2010 Jul 22;6(7):e1001035. doi: 10.1371/journal.pgen.1001035 (PMC2908705; doi:10.1371/journal.pgen.1001035)
Supplement: Table S5 — Logistic regression of clinical phenotypes on rs1801725. We report the effect size and standard error of the rs1801725 T allele from logistic regressions of each clinical phenotype. (0.04 MB DOC) [file pgen.1001035.s009.doc]

| **Phenotype** | **Beta** | **Standard Error** |
| --- | --- | --- |
| Coronary heart disease | -0.0345 | 0.0894 |
| Hypertension | 0.0193 | 0.0479 |
| Kidney stones | 0.0472 | 0.167 |
| Myocardial infarction | -0.0559 | 0.0974 |
| Osteoarthritis | -0.149 | 0.0684 |
| Osteoporosis | 0.158 | 0.0850 |
| Stroke | 0.0591 | 0.166 |
