# Supplementary material for: Genome-Wide Meta-Analysis for Serum Calcium Identifies Significantly Associated SNPs near the Calcium-Sensing Receptor (CASR) Gene
Source: PLoS Genet. 2010 Jul 22;6(7):e1001035. doi: 10.1371/journal.pgen.1001035 (PMC2908705; doi:10.1371/journal.pgen.1001035)
Supplement: Table S6 — Studies of CASR mutations and serum calcium. A survey of previous studies which investigate the relationship between CASR mutations and levels of serum calcium. (0.05 MB DOC) [file pgen.1001035.s010.doc]

| **Author** | **Year** | **N** | **Sex** | **A986S-Ca association** | **Age, mean (SD), [range]** | **Country** | **Ethnicity** | **Comment** |
| --- | --- | --- | --- | --- | --- | --- | --- | --- |
| Cole | 1999 | 163 | women | Association | [18-35] | Canada | Caucasians | participants in a prospective study |
| Cole | 2001 | 387 | women | Association | [18-35] | Canada | Caucasians |  |
| Miedlich | 2001 | 102 | men + women | No association | 55 (±12); 40 (±13) | Germany | Caucasians | healthy controls (blood donors) |
| Lorentzon | 2001 | 97 | women | Association | 16 (±2) | Sweden | Caucasians | post-menarche girls |
| Cetani | 2002 | 148 | women | No association |  | Italy | NA | healthy controls |
| Young | 2003 | 102 | women | No association | 59 (±5); 56 (±6) | New Zealand | NA | post-menopausal women part of a RCT |
| Bollerslev | 2004 | 1252 | women | No association | [70-85] | Australia | mainly Caucasians | post-menopausal women |
| Scillitani | 2004 | 337 | men + women | Association | [18-65] | Italy | Caucasians | healthy blood donors; R990G and Q1011E mutations associated with decreased serum calcium |
| Kelly | 2006 | 121 | men + women | Association | [20-60] | Scotland | Caucasians | healthy hospital staff |
| Marz | 2007 | 3259 | men + women | Association | 63 | Germany | Caucasians | individuals hospitalized for coronary angiography |
| Laaksonen | 2009 | 286 | men + women | Association | [31–43] | Finland | Caucasians | healthy adults |
